# A practical guide to RRKM theory, its simplified multi-well version AWATAR and master equation modelling of radiative processes

**DOI:** 10.1039/d6cp00705h

**Published:** 2026-04-30

**Authors:** Magdalena Salzburger, Marc Reimann, Jessica C. Hartmann, Milan Ončák, Martin K. Beyer

**Affiliations:** a Universität Innsbruck, Institut für Ionenphysik und Angewandte Physik Technikerstrasse 25 6020 Innsbruck Austria martin.beyer@uibk.ac.at

## Abstract

Unimolecular reactions and rearrangements in the gas phase under ultra-high vacuum conditions are not correctly described with equilibrium thermodynamics, since the heat bath is missing, and radiative thermalization is slow for small systems. This is the realm of Rice Ramsperger Kassel Marcus theory, commonly referred to RRKM theory, where the unimolecular rate constant is determined by the internal energy *E* of the system and the energetically accessible quantum states of the minimum and transition structures. We recently expanded classical RRKM theory to allow for a description of dissociation of entities with multiple energetically low-lying minima on the potential energy surface, called AWATAR for All Wells And Transition structures Are Relevant. This approach is used for master equation modelling of dissociation caused by radiative processes, such as black-body infrared radiative dissociation (BIRD) or infrared multiple photon dissociation (IRMPD). In the present tutorial review, we start with examples of RRKM and AWATAR calculations. Radiative processes are modelled in real time as well as by converging the population of a molecular ensemble to a stationary state. Examples for the simulation of BIRD and IRMPD experiments are shown. The program AWATAR 1.0 which is used for the simulations is made available. All examples are annotated in the SI.

## Abstract 2

This review provides a practical, step-by-step tutorial on RRKM theory and master equation modelling using the AWATAR software. It also explains the concept of AWATAR rate constants, which include all wells and all transition structures.

## Graphical abstract

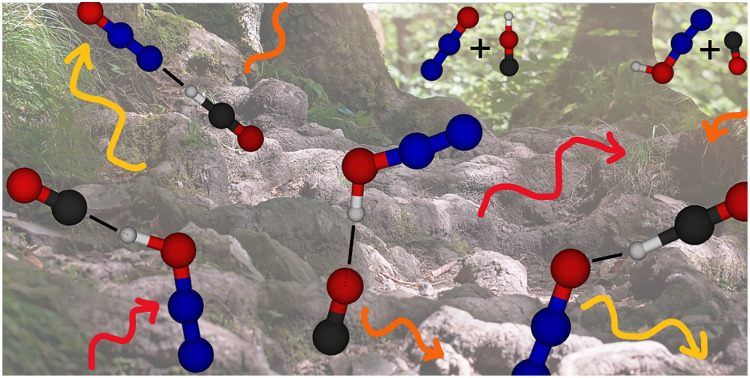

## 1. Introduction

Unimolecular isomerization and dissociation reactions in the gas phase play an important role in the description of complex processes like combustion or atmospheric chemistry.^1^ Also bimolecular gas-phase reactions are frequently described in the framework of unimolecular reactions, since their outcome is determined by the fate of the collision complex, *i.e.* back-dissociation to the reactants *vs.* radiative association^2,3^ or product formation. The energy required for a purely unimolecular reaction can be provided *e.g.* by photon absorption or energetic collisions.

Experiments on the gas phase reactivity of ions, which can be performed in mass spectrometers under well-defined conditions, are ideally suited for quantitative analysis by unimolecular statistical rate theories. Examples are collision induced dissociation (CID) experiments with the guided ion beam technique,^4^ modelling of the thermal dependence of rate coefficients in flow tube experiments,^5^ black-body infrared radiative dissociation (BIRD),^6–10^ biomolecular ion dissociation,^11–14^ dissociative photoionization pathways in the vacuum-ultraviolet (VUV) region,^15^ unimolecular decomposition of organic radical cations with potential astrochemical relevance,^16^ catalytically relevant elementary steps on metal cluster cations,^17^ enantioselective reactions in the gas phase,^18^ or potential ionic pathways in atmospheric chemistry.^19^ Fundamental insight is gained from the modelling of kinetic isotope effects,^20^ or from Rice–Ramsperger–Kassel–Marcus (RRKM) theory, which is used for example to model technologically relevant plasma processes^21^ or for the determination of the internal energy distribution in gas-phase ions.^22,23^ In infrared multiple photon dissociation (IRMPD) spectroscopy, RRKM theory has recently been employed to assess the kinetic stability of water cluster isomers.^24,25^ Programs used for RRKM calculations are, among others, Eyringpy,^26–28^ KiSThelP,^29^ Multiwell,^30^ Arkane,^31^ TUMME,^32^ MSMC,^33^ MESS,^34^ MESMER,^35^ CHIMERA,^36^ and the Zhu-Hase RRKM program.^37^

Experiments in gas-phase ion chemistry are frequently accompanied by quantum chemical calculations of the reaction potential energy surface,^38–40^ using powerful software packages such as ORCA,^41,42^ Gaussian,^43^ Molpro,^44^ Q-Chem,^45^ Turbomole,^46^ among others. When such packages are employed to calculate vibrational frequencies, their output can be used to compute thermochemical values such as enthalpy Δ*H* and Gibbs energy Δ*G*. In solution phase chemistry, especially in biochemistry, Gibbs energies are extremely useful for the description of the reaction profile, since these include entropy and provide a realistic idea of the chemical equilibrium of intermediates. However, use of Δ*G* implies constant temperature and pressure, *i.e.* a coupling of the system to an infinite heat bath, as encountered either in condensed phase or at high collision rate in the gas phase. In a gas-phase experiment under low pressure, such as the high to ultra-high vacuum of a mass spectrometer, these conditions are usually not fulfilled, and Δ*G* values are therefore not meaningful. In this case, the most useful thermochemical quantity for the description of the reaction potential energy surface is zero-point corrected energy, without applying any thermal or entropic corrections. Quantitative information on the population of specific isomers, and unimolecular dissociation or isomerization rate constants can then be derived in the framework of RRKM theory from these zero-point corrected energies and information on the accessible quantum states of the system.

This tutorial is addressed to beginners with a fair knowledge of quantum chemistry, like students working in mass spectrometry or infrared multiple photon dissociation (IRMPD) spectroscopy, who perform density functional theory (DFT) calculations to gain insight into the properties of the studied ions, possible rearrangements and dissociation pathways. The focus of this tutorial review lies on the application of RRKM theory to more complex systems, such as clusters or biomolecules, especially their unimolecular decomposition in typical experimental settings. Beyond standard RRKM calculations for individual reaction steps, we shall guide the reader to analyse multi-well potential energy surfaces, which are frequently encountered when studying biomolecular or cluster ions. To this end, we recently introduced *All Wells And Transition structures Are Relevant* (AWATAR) as a simplified multi-well extension of RRKM theory.^47,48^ On Github/Zenodo,^49^, we provide the AWATAR code which we developed for master equation modelling of BIRD experiments. The code also allows standard RRKM and AWATAR calculations, and separate calculation of the density of states of the reactant wells and sum of states of the transition structures as a function of internal energy. It also provides parameters for the real-time simulation of BIRD and IRMPD experiments. Sample input files are provided to gain hands-on experience. We hope to empower the reader to use these methodologies and adapt the examples for their own research.

## 2. Computational methods

All RRKM and AWATAR calculations are performed with AWATAR1.0, which is provided as an executable for Microsoft Windows along with the source code on Github/Zenodo.^49^ A quick start guide introduces the control of the program. All parameters and functions are described in detail in the user manual. Both documents are also available on Github/Zenodo.^49^

AWATAR1.0 requires energy and vibrational frequency calculations of local minima (Wells) and transition structures (TSs) on the potential energy surface of the system as input. The choice of the computational model, *e.g.* functional and basis set in case of a DFT calculation, is up to the reader. However, the results are in general very sensitive to relative energies and barrier heights. When discussing results of AWATAR1.0, one always has to take the uncertainty of the quantum chemical calculations into account, *e.g.* through repeating the calculations with a different functional and/or basis set. The program reads Gaussian^43^ output files directly. The output of other quantum chemical packages needs to be converted to a minimal input file, which is described in the manual.

In AWATAR1.0 and throughout this review, energies are given in cm^−1^, see Table 1 for conversion factors to kJ mol^−1^, eV and Hartree. The zero-point corrected energy of the global minimum on the potential energy surface of the system is set to 0 cm^−1^. The total energy *E* of the system is then partitioned over the internal degrees of freedom of the molecule and the potential energy of higher-lying stationary states on the potential energy surface.

**Table 1:** Energy conversion factors

cm^−1^	kJ mol^−1^	eV	Hartree
1000	11.96	0.124	0.004556
8359	100	1.036	0.03809
8066	96.49	1.000	0.03675
21 947	262.6	2.721	0.1

## 3. Unimolecular reactions

### 3.1. RRKM rate constants

#### 3.1.1. Isomerization

**Fig. 1:**
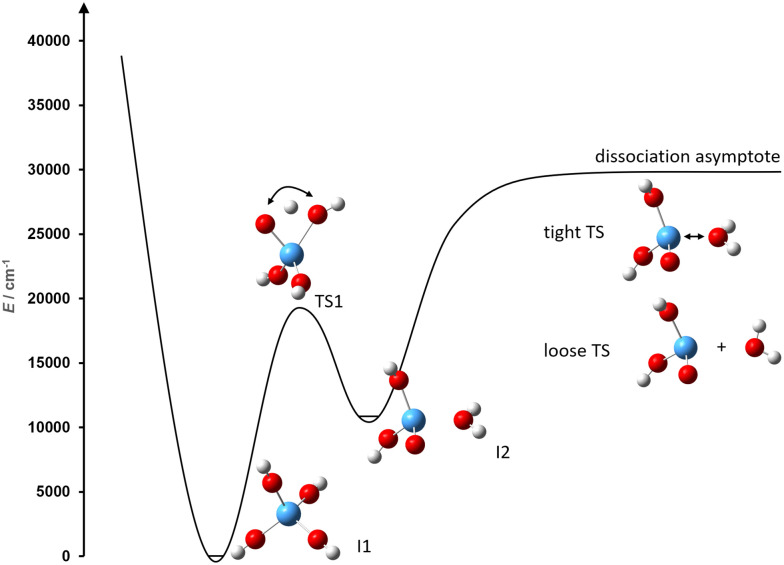
Isomerization of Ta(OH)_4_^+^*via* TS1 to TaO(OH)_2_(H_2_O)^+^, followed by loss of H_2_O, which may be represented by a tight or loose TS, calculated at the B3LYP/def2-TZVPP level of theory. See animated GIFs of TS1 and tight TS available as SI.

The description of a unimolecular reaction in the gas phase starts with the relevant stationary points on the potential energy surface (PES) of the system. Fig. 1 shows the PES of an isomerization, reaction (1), as an example.

1 Ta(OH)_4_^+^ ⇌ TaO(OH)_2_(H_2_O)^+^

In order to form a water ligand, a proton is transferred from one hydroxide ligand to another. The transition structure TS1 is the highest point on the reaction path. The motion associated with the imaginary frequency is the proton moving between reactant and product. Starting at the top, which is a first-order saddle point on the PES, the proton can move either way towards lower energy, as indicated by the arrows. An animation is provided in the SI (https://animation1.gif). If sufficient energy is available, *i.e. E* > *E*_0_(TS1), the complex will undergo the isomerization (1) forward and backward. Using the vibrational frequencies of I1, I2 and TS1, we can calculate the unimolecular rate constant for isomerization (1) in both directions by RRKM theory, using the density of states *ρ*_*i*_(*E*) of the local minimum *i* and the sum of states *N*_*y*_(*E* − *E*_0,*y*_) of TS *y* with energy *E*_0,*y*_, eqn (2).^50^2
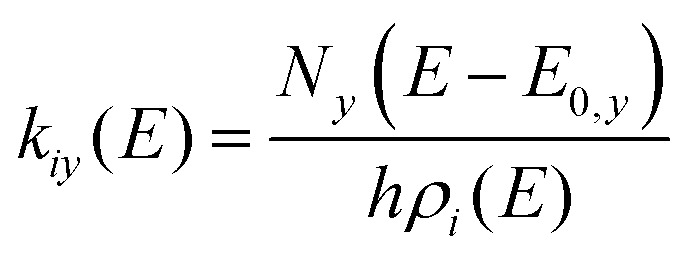
The file *example1.rrk* provides the relevant input for these calculations with AWATAR1.0. The quick start guide and the user manual describe in detail how such a file is set up, and how to work with the control buttons and the parameter panel. Panels for each well and transition structure contain the vibrational frequencies together with their infrared intensities, as well as the rotational constants of the structure and its energy in Hartree. These data were obtained by quantum chemical calculations with Gaussian, in this case at the B3LYP/def2-TZVPP level of theory. Following the standard approximation as described in detail by Gilbert and Smith,^50^ an asymmetric rotor is approximated as a symmetric top, and the rotational constants are automatically adapted by the program. This results in a 2D rotor with quantum number *J* and a 1D rotor with quantum number *K*. Due to conservation of angular momentum, *J* is constant, and only the 1D rotor is able to exchange energy with the vibrational degrees of freedom. An asymmetric rotor well therefore has (3*N* − 6) vibrational and one rotational, in total (3*N* − 5) active degrees of freedom. See SI for details on the AWATAR1.0 input and output of Example1.

Density and sum of states are numerically evaluated with the Beyer–Swinehart algorithm^51^ in the version described by Gilbert and Smith.^50^ The direct state count only works with somewhat streamlined vibrational frequencies, which must be integer multiples of an energy quantum, short BS-Quantum, defined in the parameter panel of AWATAR1.0. A typical choice for the BS-Quanrum is 1.0 cm^−1^, but small systems may require larger values to avoid unphysical behaviour. This is, because with very precise energy levels, it may be impossible to exactly match the total energy when summing up energies in the vibrational and rotational modes. A coarser graining (larger BS-Quantum) increases the number of combinatorial matches to the total energy. It is recommended to test robustness by varying BS-Quantum and ensuring that the results for the calculation of density of states and sum of states do not change significantly. See SI for a more detailed discussion of the BS-Quantum parameter.

For the density of states *ρ*_*i*_(*E*), the algorithm counts the number of ways in which the energy *E* can be distributed over the internal degrees of freedom of a well, which is then divided by BS-Quantum to yield *ρ*_*i*_(*E*) in units of (cm^−1^)^−1^. The sum of states calculation works with a similar algorithm, but it is initialized in a different way, and the algorithm compensates for different values of BS-Quantum, so that the results are largely independent of this parameter. See the book by Gilbert and Smith for details.^50^

**Fig. 2:**
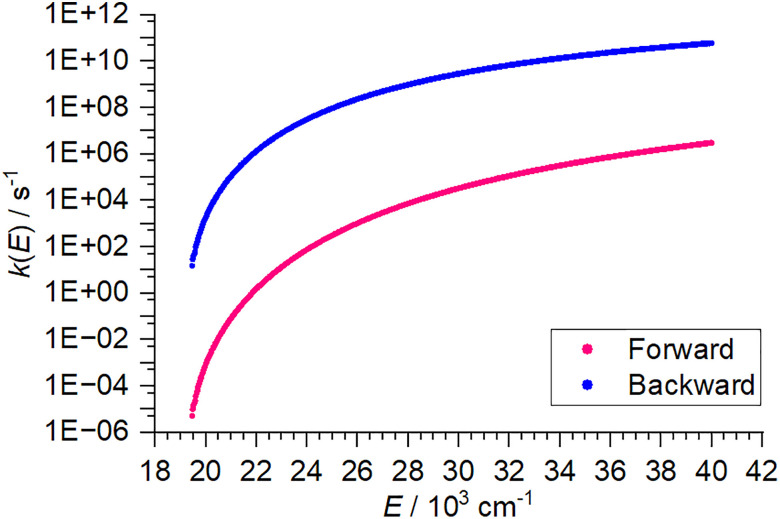
Unimolecular rate constants for the forward and backward reaction of isomerization (1).

Fig. 2 shows the RRKM rate constants *k*_*iy*_(*E*) for reaction (1), which have non-zero values only for *E* > *E*_0,*y*_. Close to the threshold, these values increase rapidly and gradually level off at higher energies. The rate constant for the backward reaction is several orders of magnitude higher than the forward reaction, indicating that the ion mostly populates the global minimum structure **I1**.

#### 3.1.2. A closer look at the density of states

The RRKM rate constants *k*_*iy*_ also illustrate the physical meaning of the density of states. If we look at the equilibrium between the two isomers **A** and **B** connected *via***TS**_**AB**_, reaction (3), we can calculate the equilibrium constant *K*_AB_(*E*) as the ratio of the rate constants of forward and backward reaction from the density of states of **A** and **B**, eqn (4), since the sum of states of **TS**_**AB**_ cancels out. Note that we still work with a microcanonical ensemble in the absence of a thermal equilibrium, therefore *K*_AB_(*E*) defined here cannot be directly linked to Δ*G*. The equilibrium constant by definition is the ratio of the probabilities *p*_**B**_(*E*) and *p*_**A**_(*E*) to find the system in isomer B and A, respectively. Essentially, this describes a chemical equilibrium situation for a single molecule that may react from isomer A to B and back, because its internal energy *E* lies above the transition structure, see Fig. 1.3
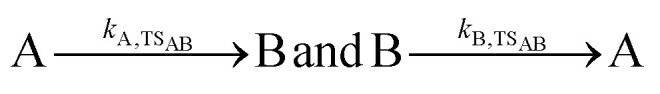
4
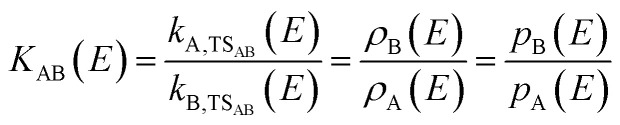
The probability *p*_**A**_(*E*) of finding the molecule in isomer **A**, is then given by eqn (5).5
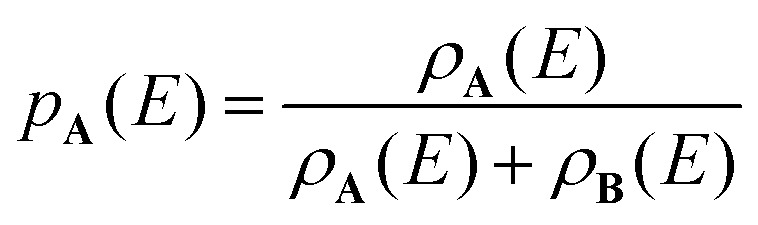
This means that comparison of the density of states of the given isomers allows to determine easily which isomer is the most probable one at a given energy. This is, especially for higher energies *E*, much more informative than the relative energies of the isomers.

**Fig. 3:**
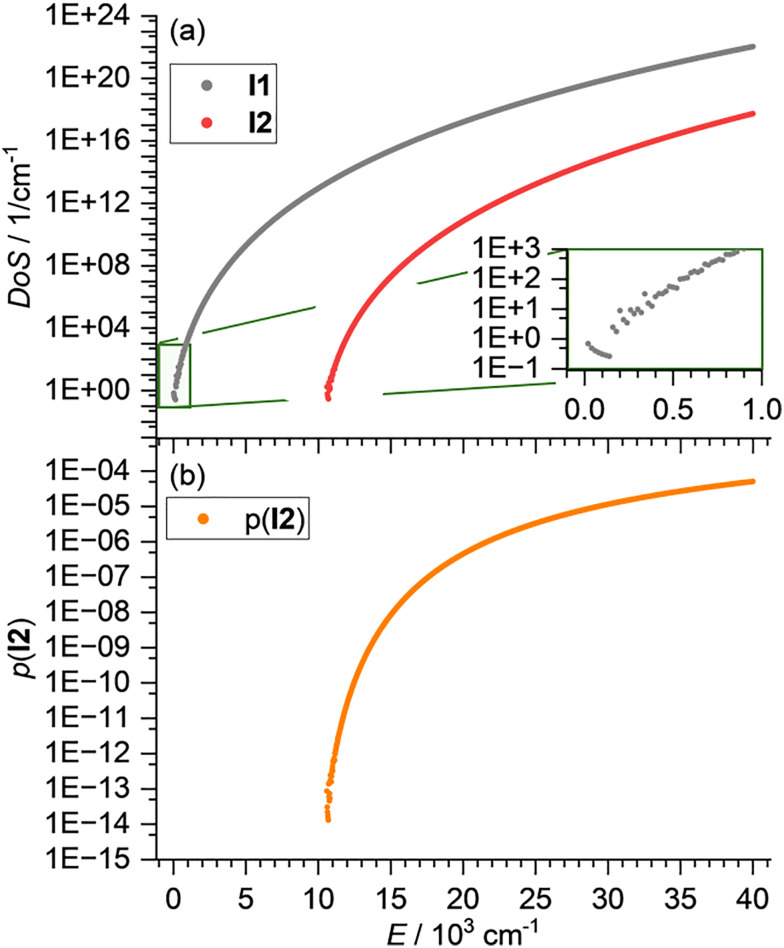
(a) Density of states of **I1** and **I2** and (b) probability to find the system in **I2** as a function of energy.

Fig. 3(a) shows the density of states of **I1** and **I2** which rapidly increases with energy, with **I1** reaching a density of states of 10^20^ (cm^−1^)^−1^ at *E* ≈ 30 000 cm^−1^. Up to *E* ≈ 1200 cm^−1^, *ρ*_**I1**_(*E*) does not increase monotonically. This does not affect the RRKM calculations here, since the barrier for isomerization (1) is close to 20 000 cm^−1^. However, if isomerization *via* low-lying transition structures is analyzed, similar numerical problems may be encountered. Fig. 3(b) shows the probability of **I2**, calculated with eqn (5). The probability for **I1**, *p*(**I1**) is always near 1 and therefore not shown.

Eqn (5) describes the situation for two isomers, but it can be generalized to any number of isomers. The probability *p*_*i*_(*E*) of finding a molecule or cluster in any of the energetically accessible isomers *i* is given by the ratio of the density of states *ρ*_*i*_(*E*) and the sum of the density of states of all isomers, eqn (6). We have used this equation to calculate the population of higher-lying isomers in cluster experiments, *e.g.* for aurosilanes^52^ or sodium chloride clusters.^47^6
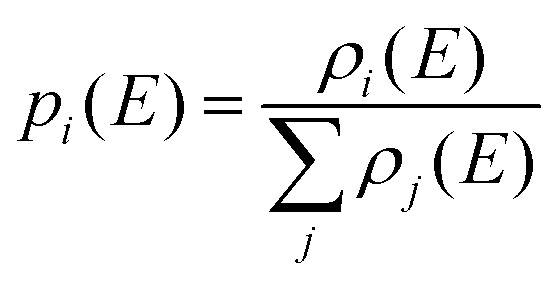


#### 3.1.3. Dissociation: tight *vs.* loose transition structure

RRKM theory is also very useful to describe dissociation reactions. In many cases, dissociation of a molecule or cluster proceeds by asymptotically reaching the dissociation threshold, thus there is no transition structure with an imaginary frequency. There are two possibilities to still model the dissociation: Using the quantum chemical calculation of the well to represent the transition structure (tight transition structure), or using a so-called loose transition structure.

The tight transition structure requires specification of the dissociation energy in the parameter panel, and substituting the reaction coordinate with a suitable vibrational mode. To this end, one visually inspects the vibrational modes, *e.g.* with GaussView,^53^ Molden^54^ or ChemCraft,^55^ and identifies the dissociative mode. In general, this is a stretching mode where the separating fragments are oscillating against each other with significant amplitude. For TaO(OH)_2_(H_2_O)^+^, this is illustrated in Fig. 1 and animation2.gif available as SI. In AWATAR1.0, the frequency calculation of the well can be read both as a well and as a tight transition structure, and the dissociative mode is removed from the list of frequencies of the TS. In addition, the TS energy must be adjusted to reflect the dissociation energy. See SI for details on the implementation in *example2.rrk*.

The tight transition structure model requires the identification of a realistic reaction coordinate. In the dissociation of Ta(OH)_4_^+^, the molecular ion must first rearrange to TaO(OH)_2_(H_2_O)^+^, which means we have to describe the dissociation in two steps, rearrangement followed by dissociation. In addition, one can expect that the vibrational degrees of freedom which correspond to motions of the two fragments relative to each other, such as bending modes, soften significantly when the interaction gets weaker. One could account for this by arbitrarily changing the frequencies of suitable modes, but this adds additional uncertainty to the result.

These problems are resolved using the loose TS model. Here, the fragments are treated as fully separated, which allows them to rotate independently of each other. In the general case of non-linear polyatomic fragments, five degrees of freedom that are vibrations in the tight transition structure model become rotations, see Table 2 and the discussion in Section 3.1.1. This leads in general to a substantial increase in the sum of states, and therefore a higher RRKM rate constant. The implementation of the loose TS model in AWATAR1.0 follows the procedure described by Rodgers, Ervin and Armentrout.^56^ For dissociation of metal–ligand complexes or weakly bound systems like water clusters, the loose TS model is usually preferred. Fig. 1 shows the loose transition state for water loss from TaO(OH)_2_(H_2_O)^+^.

**Table 2:** Number of active degrees of freedom for a non-linear well and non-linear fragments

Active degrees of freedom	Well	Tight TS	Loose TS
Vibrational	3*N* − 6	3*N* − 7	3*N* − 12
Rotational	1	1	6
Sum	3*N* − 5	3*N* − 6	3*N* − 6

**Fig. 4:**
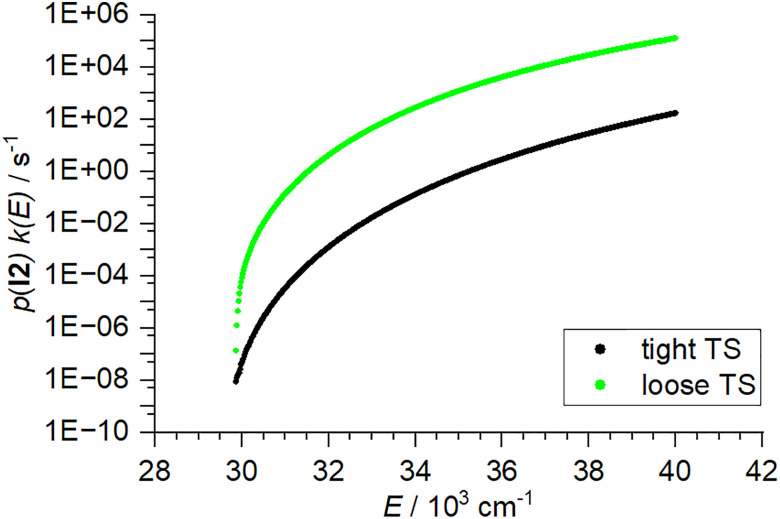
Comparison of tight and loose transition structure model for the loss of water from Ta(OH)_4_^+^*via* isomerization to TaO(OH)_2_(H_2_O)^+^.

The RRKM rate constants for water loss from TaO(OH)_2_(H_2_O)^+^ are multiplied with the probability that this energetically higher-lying isomer is actually populated, eqn (6). Fig. 4 compares the resulting unimolecular rate constants for water loss modelled with the tight and loose TS models, the corresponding AWATAR1.0 file is *example2.rrk*. The loose TS model consistently predicts significantly higher dissociation rate constants, which are in general considered to be more realistic. The tight TS model can be tweaked empirically to yield higher rates by deliberate modification of vibrational frequencies. A more elaborate approach to describe unimolecular dissociation is variational transition state theory,^57^ which requires significant additional computational effort.

A technical note on the description of rotations: to keep the calculations manageable, all non-linear molecules are treated as symmetric tops by AWATAR1.0. Upon reading the two TS fragments with buttons “Load loose TS Fragment 1” and “Add loose TS Fragment 2”, AWATAR1.0 automatically calculates the geometric average of the two most similar rotational constants when reading the quantum chemical results, and employs 1D and 2D rotor models as described by Gilbert and Smith.^50^

### 3.2. AWATAR rate constants

In the tight and loose transition structure models described above, we discussed rearrangement of the system prior to dissociation. We have seen that we can calculate the probability of finding a molecule or cluster in a specific isomer *via*eqn (6). The effective RRKM rate constant for dissociation of Ta(OH)_4_^+^ by loss of H_2_O was calculated from the unimolecular rate constant of TaO(OH)_2_(H_2_O)^+^, **I2**, multiplied by the fraction of ions that have this structure at a given time.

More generally speaking, when we combine eqn (2) with eqn (6) to calculate the effective rate *k*_eff*,y*_(*E*) of a unimolecular reaction of the system from a specific well *i via* a specific TS *y*, we see immediately that the density of states of the specific well *i* cancels out, leaving the sum of the density of states in the denominator, eqn (6). This is an extension of the key idea of RRKM theory that the rate constant is determined by the number of accessible quantum states above the transition structure and the density of quantum states in the minimum. In situations where isomerization between isomers is fast compared to reaction *via* the transition structure, the sum of the density of states determines the probability of the reaction.7
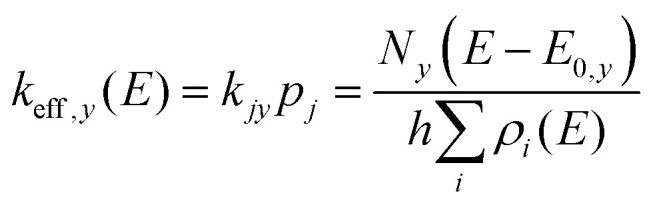
This illustrates that all energetically accessible wells are relevant in the calculation of a unimolecular rate constant. However, similar arguments apply to the transition structures: If a product or set of products can be reached *via* distinguishable transition structures, each and every one of them will contribute to the unimolecular rate constant. We therefore introduced the AWATAR rate constant,^47^eqn (8), where we calculate the rate constant *k*_AWATAR_(*E*) by adding the sum of states *N*_*y*_(*E* − *E*_0,*y*_) of all transition structures *y* that lead to a set of products, and divide by the sum of the density of states *ρ*_*i*_(*E*) of all energetically accessible wells *i*. A set of products is *e.g.* a fragment ion observed in a photodissociation experiment, together with a neutral fragment, both of which may be represented by a variety of isomers.8
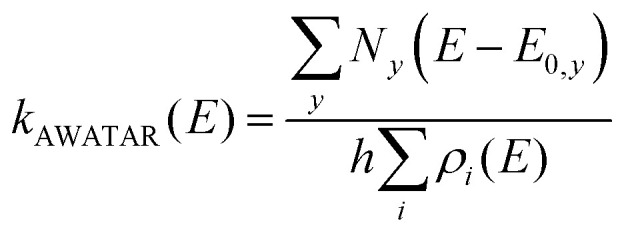
AWATAR is especially advantageous for clusters with energetically low-lying isomers. It was successfully employed for the explanation of the magic behavior of (NaCl)_4_Na^+^ and in the master equation modelling of BIRD experiments with small water clusters.^47,48,58^

**Fig. 5:**
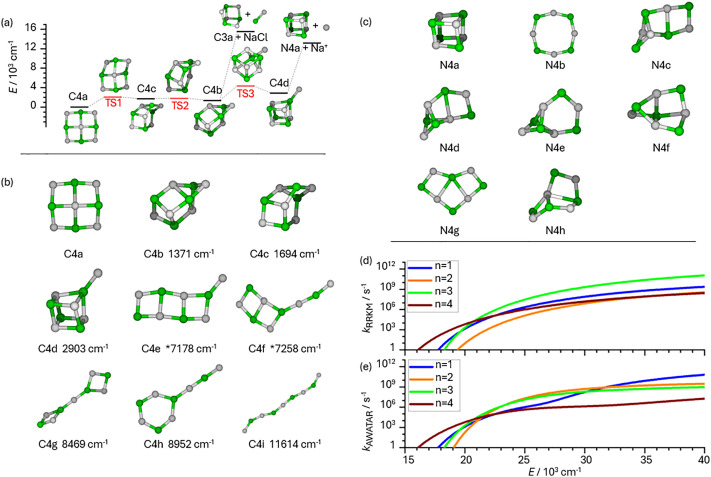
(a) Potential energy surface (PES) of (NaCl)_4_Na^+^, including transition states for rearrangements between energetically low-lying isomers C4a–C4d and loss of NaCl or Na^+^, calculated at the CCSD//B3LYP-D3/aug-cc-pVDZ level of theory. (b) Isomers of (NaCl)_4_Na^+^ found with quantum chemical calculations together with their relative energies, calculated at the CCSD/DZ//B3LYP-D3 level of theory. Energies marked with * calculated at the CCSD/DZ//ωB97XD/aug-cc-pVDZ level, since these isomers are saddle points on the B3LYP-D3/aug-cc-pVDZ level. (c) Isomers of neutral fragment (NaCl)_4_ used for the loose TSs that describe loss of Na^+^ in AWATAR. (d) RRKM rate constants *k*_RRKM_ for dissociation *via* loose TSs from well C4a, using the global minima for all fragments. (e) AWATAR rate constants *k*_AWATAR_ for the dissociation of (NaCl)_4_Na^+^ according to reaction (9). Panels (a)–(c) are adapted from ref. 47 under a CC-BY License. © 2024 The Authors.

The effect of AWATAR rate constants is illustrated with the dissociation of (NaCl)_4_Na^+^, *example3.rrk*. The potential energy surface with the lowest lying isomers is shown in Fig. 5(a). As isomerization is fast (see Fig. S1 in the SI), this system should be treated with the AWATAR approach for more accurate results. The file *example3.rrk* is quite complex, since it contains all 9 isomers of (NaCl)_4_Na^+^, which we found in our quantum chemical calculations, as wells *i*, see Fig. 5(b) and original work for details.^47^ Experimentally, we observed only stoichiometric dissociation of the salt clusters, *i.e.* loss of (NaCl)_*n*_ units, with *n* = 1–4, reaction (9).

9 (NaCl)_4_Na^+^ → (NaCl)_4−*n*_Na^+^ + (NaCl)_*n*_ *n* = 1–4

Not only the reactant clusters occur in different isomers, also the fragments may be formed in any of the energetically accessible isomers. Fig. 5(c) shows the isomers of (NaCl)_4_ which together with Na^+^ describe the loose TSs for the *n* = 4 channel of reaction (9). Each combination of fragment isomers contributes to the sum of states in a loose transition structure model, therefore all combinations of isomers of stoichiometric neutral and charged fragments must be considered.

In the experiment, all fragment cluster sizes (NaCl)_4–*n*_Na^+^, *n* = 1–4, are observed. Therefore, we were interested in the unimolecular rate constants for dissociation into the different fragment cluster sizes, and defined four AWATAR reaction channels as dissociation from all wells into all combinations of fragment isomers for a given fragment cluster size *n*. The resulting AWATAR dissociation rate constants are shown in Fig. 5(e), rate constants obtained with a single-well loose TS model are shown in Fig. 5(d) for comparison. The curves of the AWATAR rate constants for the individual fragment sizes are qualitatively different from Fig. 5(d): the fragments (NaCl)_3_Na^+^ and (NaCl)_4_ have relatively low-lying isomers with low-frequency vibrational modes, generating a high sum of states. As a consequence, the AWATAR rate constant increases dramatically for *n* = 1 around 3.3 eV, and the *n* = 4 rate constant exhibits a more moderate increase around 3.9 eV. This illustrates that also all energetically accessible isomers of product fragments, which constitute different loose transition structures, contribute to the rate constant, underlining that All Wells And Transition structures Are Relevant (AWATAR). The AWATAR rate constants in this example explain the magic behavior of (NaCl)_4_Na^+^: the Na^+^ loss channel has a relatively moderate barrier, but the rate constant for dissociation is still relatively small, because the loose transition states for this channel have a small sum of states.

### 3.3. Describing ion–molecule reactions with unimolecular rate theory

RRKM and AWATAR calculations can be very helpful in the analysis of ion–molecule reactions in the gas phase at low pressure. The idea is that an ion–molecule reaction A^+^ + B starts with the formation of a collision complex (AB)^+^*, reaction (10), with a rate constant *k*_c_. The internal energy of the collision complex (AB)^+^* consists of the binding energy A^+^ − B and the internal energy of the reactants and their relative kinetic energy before the collision. (AB)^+^* either undergoes back-dissociation with *k*_b_, reaction (11), or reacts to products *via* one or more transition structures, with a rate constant *k*_p_, reaction (12). RRKM or AWATAR calculations can be applied to calculate unimolecular rate constants *k*_b_ and *k*_p_ that describe the fate of the collision complex. Combined with a realistic model for the collision rate *k*_c_ and the probability *p*(*E*) that the collision complex is formed with energy *E*, this amounts to a prediction of the ion–molecule reaction rate coefficient *k*_abs_ from first principles, eqn (13), provided the collision complex actually forms at the rate predicted by the collision model and dynamic effects do not play a significant role. Also effects of angular momentum conservation, discussed *e.g.* by Aristov and Armentrout,^59^ are not included in this treatment.10
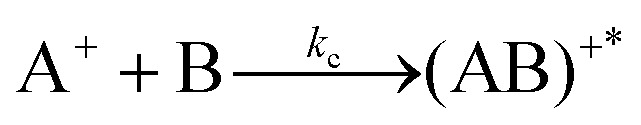
11
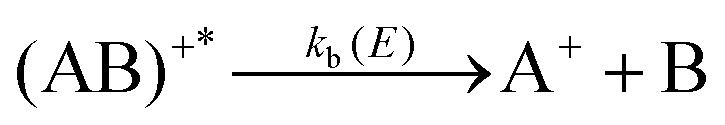
12
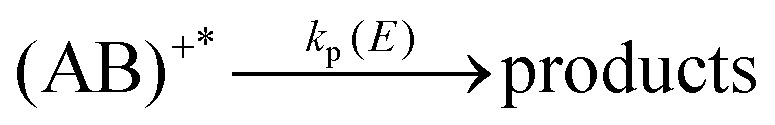
13
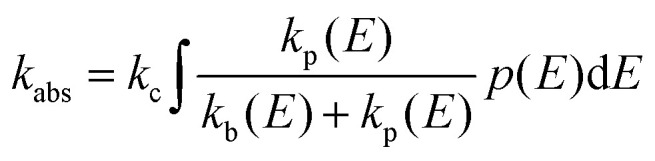
The calculation of the unimolecular rate constants *k*_b_(*E*) and *k*_p_(*E*) in AWATAR is demonstrated using the example of the proton transfer reaction from OCH^+^ to N_2_O, reaction (14).

14 HCO^+^ + N_2_O → CO + N_2_OH^+^

**Fig. 6:**
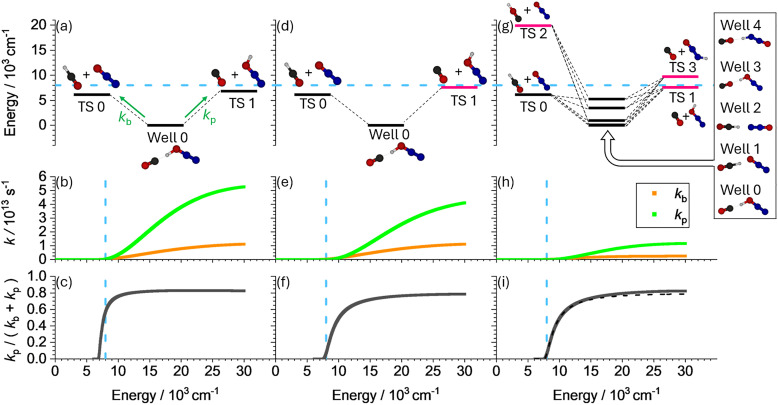
PES of proton transfer reaction from OCH^+^ to N_2_O, calculated at the ωB97X-D4/def2-QZVPP level of theory (upper panels), rate constants (middle panels), and ratio of product formation (lower panels) as a function of energy *E*. *E* = 8000 cm^−1^ is marked with a dashed blue line. (a)–(c) Energies as calculated. (d)–(f) Energy *E* of TS1 (pink line) adapted such that the difference to TS 0 corresponds to the difference of experimental proton affinities.^60^ (g)–(i) AWATAR treatment including higher lying isomers of wells and transition structures. In (g) Well 1 and Well 0 are close in energy, represented by the same horizontal line. In (i), the dashed line is the curve from (f) to illustrate the difference.

In the file *example4.rrk*, all higher-lying isomers are neglected, using the PES shown in Fig. 6(a). The starting point of the reaction, HCO^+^ + N_2_O, is modelled by the loose transition structure TS 0, the collision complex is Well 0, and the products CO + N_2_OH^+^ are represented by TS 1. Accordingly, two reaction channels are defined: Channel 0, which represents the pathway from Well 0 to TS 0, *i.e.* back-dissociation, and Channel 1, which represents the pathway from Well 0 to TS 1, *i.e.* product formation. These channels are used to calculate *k*_b_ and *k*_p_, respectively, for all energies. Note, that the zero of the energy scale is set to the energy of Well 0. However, as discussed earlier, the available energy in the system is always higher, because it includes the complex formation energy, the internal energy of the reactants, and their relative kinetic energy prior to the formation of the collision complex. Radiative association is not considered in this calculation.

For example, consider an energy of 8000 cm^−1^, indicated by the blue dashed line in Fig. 6. This value is slightly above the highest transition state, which ensures that product formation is energetically possible. Fig. 6(b) shows *k*_b_ and *k*_p_, while Fig. 6(c) shows the fraction 
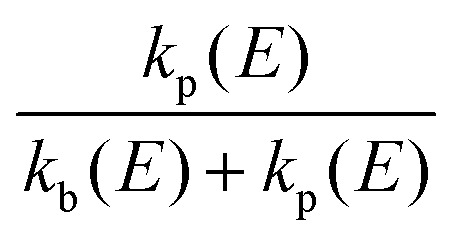
. At 8000 cm^−1^, this fraction is 0.6, meaning that 60% of collisions with this energy will lead to product formation.

This calculation is highly sensitive to the relative energies of the transition structures. Experimental proton affinities,^60^ which are determined with high accuracy, suggest that the enthalpy difference between the transition structures is 1572 cm^−1^. Using the calculated thermal corrections to convert between enthalpy and zero-point corrected energy, this results in an energy difference of 1438 cm^−1^. To reflect the experimentally established thermochemistry, we adjusted the absolute energy of TS 1 to 7555 cm^−1^, ensuring that the energy difference between TS 0 and TS 1 is 1438 cm^−1^, see *example5.rrk* and Fig. 6(d). Note, that in this case, only the energy difference between the transition structures is relevant, as eqn (15) shows that the density of states cancels out in eqn (13).15
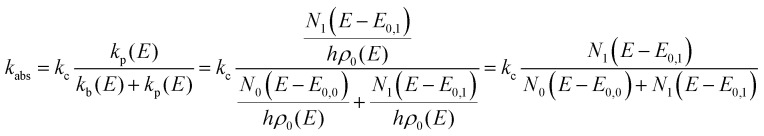
Equivalent results are obtained by adjusting the energy of TS 0 instead of TS 1, provided the available energy is also adjusted accordingly. The main challenge is to determine the available energy relative to the lowest-lying TS. The PES, the rate constants, and the fraction 
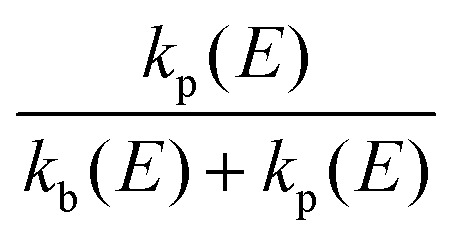
 are shown in Fig. 6(d)–(f), respectively. With these empirically adjusted numbers, product formation is less probable, occurring with a probability of 7% at an available energy of 8000 cm^−1^. Since only a fraction of the collisions occurs with sufficiently high energy, the overall probability of product formation across all energies is even smaller. Comparison with known reaction rate coefficients shows, that this result is qualitatively consistent with experimental findings, as an estimate of the magnitudes, using the collision rate (low 10^−9^ cm^3^ s^−1^) and the rate constant (low 10^−12^ cm^3^ s^−1^),^61,62^ yields only about 0.1% of collisions experimentally lead to product formation.

To determine the overall probability of product formation upon collision quantitatively, the energy-dependent results need to be convoluted with the thermal energy distribution *p*(*E*) of the collision partners, eqn (13). However, such a feature is not implemented in AWATAR1.0.

Next, we test whether the AWATAR eqn (8) leads to different results. In the file *example6.rrk*, we included COH^+^ and HN_2_O^+^ as additional isomers of reactant and product, and several isomers of the collision complex, as shown in Fig. 6(g). The TS energies are determined relative to TS 0 using experimental proton affinities, and the channel definitions are adapted to include all isomers. The PES, the rate constants and the resulting fraction 
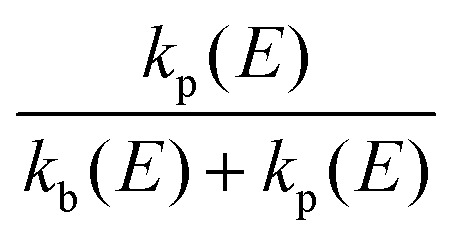
 are shown in Fig. 6(g), (h) and (i), respectively. While the rate constants *k*_p_(*E*) and *k*_b_(*E*) are noticeably modified, the fraction 
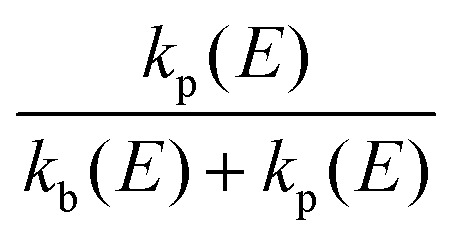
 again does not change significantly compared to Fig. 6(f), see dotted line in Fig. 6(i). In this case, the density of states of the Wells cancels out, and the higher-lying transition structures do not contribute much to the sum of states due to their relatively high energy.

## 4. Master equation modelling of radiative processes

As discussed earlier, the internal energy of molecular clusters or molecular ions is a critical parameter in processes such as dissociation. In most mass spectrometric experiments, the internal energy of the system is determined by energetic or thermal collisions, depending on the kinetic energy of the ions. Collisional energy transfer is, however, currently not implemented in the AWATAR program, since it was specifically developed for the modelling of BIRD experiments, typically performed in FT-ICR instruments at pressures *p* < 10^−9^ mbar.^63^ At such low pressures, with collision rates ≲0.1 s^−1^, radiative energy transfer is usually dominant. Trapped ions exchange IR photons with the environment. After a sufficient trapping time at constant temperature, the system will equilibrate with the environment, if thermal energy is not sufficient for dissociation. The internal energy distribution in this case is simply a Boltzmann distribution.

For more complex scenarios, the time evolution of the internal energy distribution can be simulated using Master Equation Modelling (MEM). AWATAR1.0 enables MEM simulations for various processes relevant in ultra-high vacuum conditions, such as the cooling or warming of molecular populations, changes in internal energy following absorption of a single photon, or the effects of exposure to a constant photon flux (*e.g.*, from an infrared laser in an IRMPD experiment). Additionally, MEM in AWATAR1.0 can account for the loss of high-energy molecules due to dissociation and calculate corresponding MEM dissociation rate coefficients. As noted above, AWATAR1.0 has been developed for the simulation of radiative processes in an FT-ICR mass spectrometer under ultra-high vacuum conditions, therefore collisions are not taken into account.

Here, MEM is discussed qualitatively using simple examples, to provide an intuitive understanding of the simulation. A detailed description of the equations describing photon absorption and emission as well as unimolecular dissociation in MEM as implemented in AWATAR1.0 can be found in the SI. For information on how further scenarios can be treated using AWATAR1.0, see the user manual, and our publications.^48,58,64^

### 4.1. Real-time MEM of radiative energy transfer without dissociation

In real-time MEM mode, as opposed to steady state MEM described below, AWATAR1.0 simulates the evolution of the population over a predefined period of time in finite time steps Δ*t*.

**Fig. 7:**
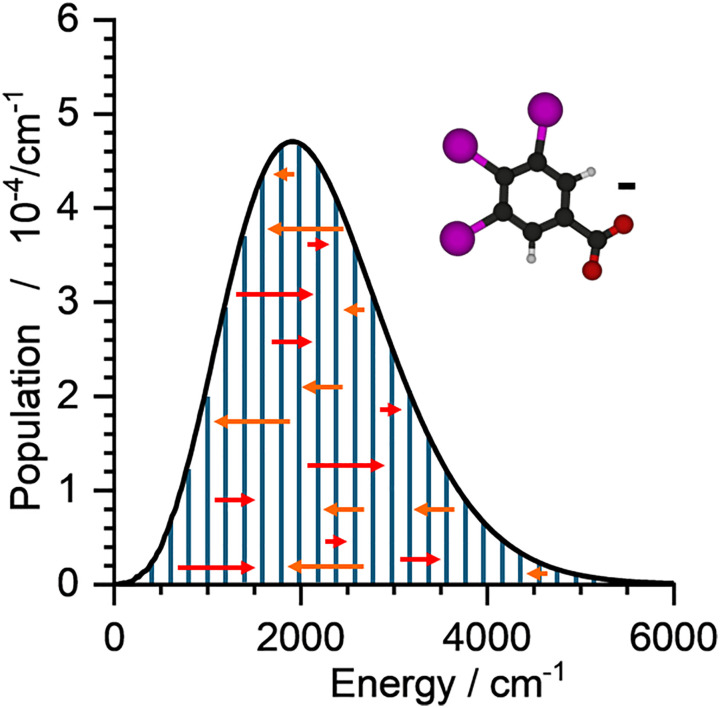
Single well without dissociation: population for C_6_H_2_I_3_COO^−^ at 300 K in stationary state. Red arrows indicate photon absorption, orange arrows photon emission. Both processes shift population from one energy bin to another. In the stationary state, these shifts exactly cancel out.

In this chapter, the evolution of the internal energy of a population of C_6_H_2_I_3_COO^−^ molecules in four different scenarios is simulated: (1) cooling from high temperature, (2) heating from low temperature, (3) cooling after absorption of an infrared photon, and (4) heating due to a constant photon flux. This specific molecular ion, shown in Fig. 7, was chosen because it neither isomerizes nor dissociates at the energy available in the simulations. Due to the heavy iodine atoms, it has some low-frequency vibrational modes, resulting in an appreciable density of states that minimizes numerical problems.

Fig. 7 illustrates how photon absorption and emission modify the population over time, shifting parts of the population to higher and lower energies, respectively. If the initial population differs from thermal equilibrium, these processes will gradually transform the population to a Boltzmann distribution. MEM enables us to understand how fast this takes place for a specific system, and AWATAR1.0 covers a variety of typical experimental situations.

We start with a scenario that represents thermalization of ions that emerge from a heated electrospray capillary and are trapped under collision-free conditions at room temperature in an FT-ICR mass spectrometer. The file *example7.rrk* simulates the gradual equilibration of a population of C_6_H_2_I_3_COO^−^ molecular ions with an initial temperature of 500 K, in a room temperature (300 K) black-body radiation field.

**Fig. 8:**
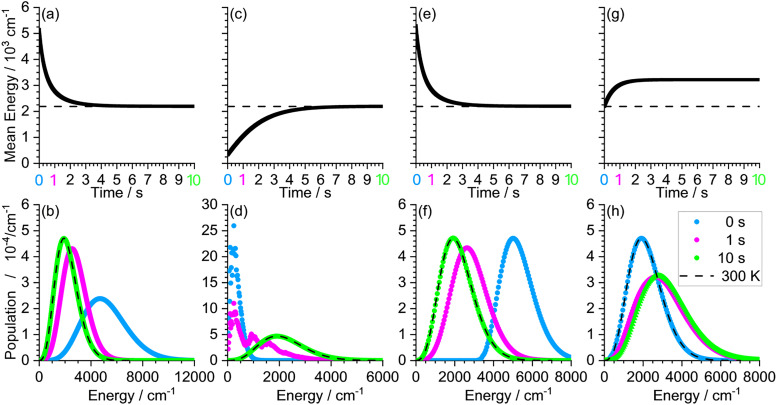
MEM of C_6_H_2_I_3_COO^−^ simulating changes in internal energy in a 300 K environment: (a) and (b) cooling of an initial 500 K population; (c) and (d) heating of an initial 100 K population; (e) and (f) equilibration of a 300 K population after absorption of a 3091.43 cm^−1^ photon; (g) and (h) IRMPD experiment with absorption of 1694.44 cm^−1^ photons at a rate of 1 photon per second.

For the numerical simulation, the energy range is divided into finite energy bins. At *t* = 0, the population is initialized with a Boltzmann distribution at 500 K, shown in blue in Fig. 8(b). Upon simulation, photon absorption and photon emission shift small fractions of the population of each energy bin towards bins at higher and lower energies, respectively. The rates for absorption and emission are calculated for each energy bin using Einstein coefficients calculated from frequency and intensity of the computed harmonic vibrational frequencies.^64^ With this information, the change of the population within a finite time step Δ*t* can be determined.

The results of this MEM calculation are shown in Fig. 8(a) and (b). Fig. 8(a) shows how the mean energy, which is initially more than 5000 cm^−1^, decreases with time, approaching the 300 K value indicated with a dashed line within a few seconds. Fig. 8(b) shows the corresponding populations *t* = 1 s and *t* = 10 s. Photon emission is initially more likely than photon absorption, so the population shifts towards lower energies. After 1 s, (shown in pink in Fig. 8(b)), the population is already near the 300 K distribution (shown with a dashed line), while after 10 s, the population is fully equilibrated (shown in green).

The file *example8.rrk* simulates heating of an initially cold population, generated *e.g.* in a liquid-nitrogen cooled ion source, with an initial temperature of 100 K. The results are shown in Fig. 8(c) and (d). As expected, the mean energy rises within a few seconds, and after 10 s the population is fully equilibrated. However, the population after 1 s (shown in pink) does not resemble a Boltzmann distribution, since it features some local maxima, the most pronounced ones at approximately 240 cm^−1^ and 980 cm^−1^. To understand these features, it is instructive to have a more detailed look on how the shift of the population is calculated: At *t* = 0 the population is Boltzmann distributed as shown in blue in Fig. 8(d). Each data point represents one energy bin. To calculate the change of the population of one of these bins within the next time step, the total population losses and gains are needed. The losses are calculated *via* the absorption or emission rates for all possible transitions to other bins. This is done using the information provided by a quantum chemical frequency calculation and summarized in the panel of the well in the AWATAR program: The energies of the vibrational modes determine which transitions are possible. The probability for those transitions is described by the Einstein coefficients for spontaneous emission and by Planck's law of radiation for stimulated emission and absorption in a black-body radiation field. The Einstein coefficients for a specific vibrational mode are a function of the frequency and the infrared intensity obtained by quantum chemical calculations.

Now let's turn back to the question, where the features in the pink population in Fig. 8(d) come from: A closer examination of the vibrational modes in the panel “Well 0” shows, that for most vibrational modes the infrared intensities are small, but in the range between 680 cm^−1^ and 820 cm^−1^ some vibrational modes have relatively high intensity. This means, that the original population will be shifted preferentially by photons exciting those vibrational modes, by 684 cm^−1^, 707 cm^−1^, 773 cm^−1^ or 816 cm^−1^, causing the second peak. The shoulder on the high energy side is caused partly by absorption of more than one photon, and partly by absorption of photons with a higher energy. The latter can be seen by setting the intensity of all higher energy vibrational modes to zero, and re-running the simulation for comparison (results not shown). In Fig. 8(c), which displays the results without such modifications, we see that the internal energy equilibrates within 6 s, and the population has again reached the 300 K Boltzmann distribution after 10 s, shown in green in Fig. 8(d).

The file *example9.rrk* describes the evolution of the population after absorption of a photon with *hν* = 3091.43 cm^−1^, corresponding to the energy of the C–H symmetric stretch vibration. The results are shown in Fig. 8(e) and (f). In this scenario, the population is initialized at *t* = 0 with a Boltzmann distribution at 300 K, which is shifted to higher energies by the energy of the absorbed photon (shown in blue in Fig. 8(f)). As can be seen in Fig. 8(e), after 1s, a part of the photon energy is still in the molecule, while at about 3 s, the population is already very close to 300 K.

Finally, the file *example10.rrk* simulates the influence of a constant photon flux as provided, *e.g.*, by an infrared laser in an IRMPD experiment. The photon energy corresponds in this example to the frequency of the COO antisymmetric stretching mode (1694.44 cm^−1^). The rate of photon absorption is set to a fixed value. If needed, an individual rate can be set for each well (see user manual). Here we have only one well, and the rate is set to 1 s^−1^. The results are shown in Fig. 8(g) and (h). Due to the additional photon flux, the mean energy initially increases. Within 2 s, a new stationary state is reached with a higher mean energy, since also the emission rates go up. The 10 s population (green) is not significantly different from the 1 s population (pink). As one might expect, a higher rate of photon absorption leads to a higher mean energy, shown in Fig. S2 in the SI.

### 4.2. Real-time MEM of IRMPD in a black-body radiation field with dissociation

For molecules that dissociate at internal energies that are non-negligibly populated, the situation becomes slightly more complex. In addition to photon absorption and emission, also dissociation is included in MEM for all bins above the energy of the lowest-lying transition structure. Therefore, it is now necessary to define reaction channels and transition structures so that AWATAR can calculate the relevant rate constants *k*(*E*). Dissociation results in the loss of part of the population, so with ongoing simulation time, the intensity of the parent molecule or cluster decreases.

Since dissociation can also be measured experimentally, for example by using a mass spectrometer, it is possible to compare simulated kinetics with experimental results. By fitting the simulated kinetics to experimental kinetics measured at different temperatures, unknown AWATAR parameters, like the rate of photon absorption in an IRMPD experiment, can be determined.

**Fig. 9:**
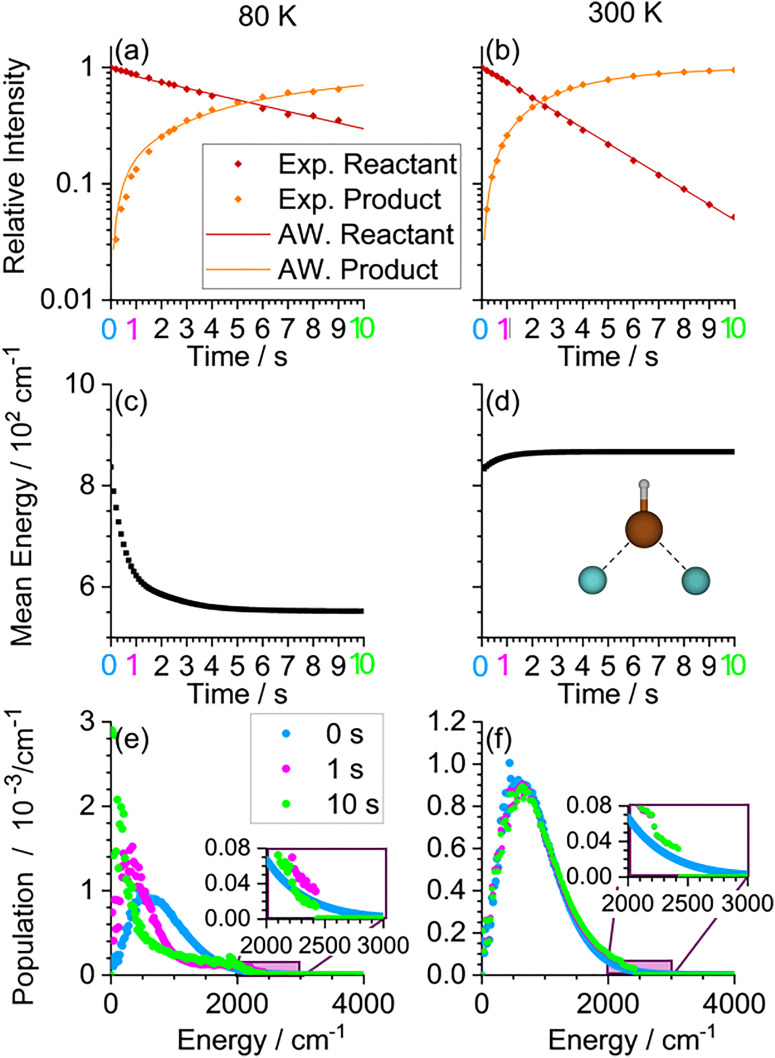
MEM of Ar_2_FeH^+^ reproducing experimental IRMPD kinetics trapped at 80 K (a), (c), (e) and 300 K (b), (d), (f). The initial cluster temperature is set to 300 K in both cases, reflecting the temperature of the ion source. (a) and (b) Experimental kinetics (circles) and simulation (lines); (c) and (d) evolution of the mean internal energy; (e) and (f) population at 0 s (Boltzmann distribution without dissociation), and after 1 s and 10 s simulation time.

In this chapter, real time MEM is used to simulate IRMPD kinetics of Ar_2_FeH^+^, which have already been experimentally measured and published in ref. 65. The molecule, shown in Fig. 9(d), has only one stable isomer and decays by losing an argon atom. The energy required for loss of an Ar atom was calculated to be 2423 cm^−1^.^65^

The file *example11.rrk* simulates the experimental scenario, using a photon energy of 1855 cm^−1^, which corresponds to the Fe–H stretching vibration, and environmental temperatures of 80 K and 300 K. The scaling factor for infrared intensities is set to 4.797, as this allows to reproduce the experimental BIRD kinetic published in ref. 65. The rate of photon absorption of 0.34 s^−1^ was determined by fitting the MEM simulation results to the experiment in the 300 K scenario. The cluster population was initialized with 300 K, the temperature of the ion source, for both environmental temperatures considered.

The resulting kinetics (lines) are shown alongside the experimental kinetics (circles) in Fig. 9(a) and (b). The fits almost perfectly reproduce both kinetics with one set of fit parameters. For a more reliable determination of the parameters, additional experimental kinetics at other temperatures would be necessary to verify whether the chosen parameter set is valid over a wider range of conditions.

Fig. 9(c) and (d) show the mean energy for each simulation. Initially, the mean energy is the same, as both start with an initial cluster temperature of 300 K determined by the temperature of the laser vaporization source. In the 80 K scenario, radiative cooling initially dominates over the influence of the infrared laser. In the 300 K environment, the mean energy increases as expected in an IRMPD experiment, where the initial cluster temperature matches the environmental temperature. Panels (e) and (f) show the populations at three selected times. At 80 K, the decrease of the mean energy is also reflected in the populations. At 300 K, the populations remain very similar over 10 s simulation time. Notably, in both cases, the high energy tail of the Boltzmann distribution is reduced above the energy of the transition structure (2423 cm^−1^). This rapid fall-off reflects the loss of population due to dissociation. The population at *t* = 0 s is the Boltzmann distribution with which the simulation starts. This starting population does not include dissociation yet and is therefore not reduced above the energy of the TS. Note that the populations do not reflect the overall loss of signal due to dissociation, they are all normalized to 100%.

### 4.3. Real-time MEM of BIRD involving multiple isomers

AWATAR can be used to perform MEM for systems with multiple isomers, provided that isomerisation occurs much faster than dissociation. When multiple isomers are included, the population of each energy bin is redistributed among all isomers according to their density of states after each time step. If several transition structures are involved, dissociation is allowed *via* all energetically accessible transition structures.

The different channel definitions allow to include all wells and all transition structures (TS) within a single channel (AWATAR approach), or to define several channels, each linking a single well to a single transition structure. As used for example in ref. 48, it is also possible to model dissociation into multiple products by defining separate channels for each product. In this case, each channel connects all wells to the transition structures that lead to the respective product (see manual for details).

**Fig. 10:**
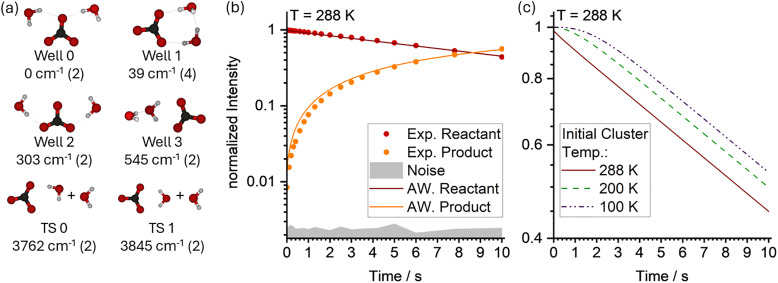
MEM simulation of BIRD kinetics of CO_3_(H_2_O)_2_^−^. (a) Optimized structures of wells and transition structures with relative energies in cm^−1^ and degeneracy in parentheses; (b) comparison of the simulated data (lines) with experimental kinetics data (circles); (c) simulation of the kinetics with different initial cluster temperatures, leading to an induction delay.

Performing MEM with multiple minima is straightforward in AWATAR: Besides loading all wells and transition structures, it works the same as with a single well and single transition structure. The file *example12.rrk* is set up to simulate BIRD kinetics of doubly hydrated carbonate radical anions CO_3_(H_2_O)_2_^−^ at an environmental temperature of 288 K. Four wells and two loose TSs are included, shown in Fig. 10(a) together with relative energies and degeneracies. Degeneracies are determined by multiplying the number of optical isomers with the spin multiplicity (see user manual for details). In this case, the degeneracy of 2 results from the spin multiplicity, which is for Well 1 multiplied with 2, the number of optical isomers of this well. The activation energy of the lowest-lying transition structure is set to 3762 cm^−1^, as determined by ref. 64. IR intensities are scaled by a parameter also taken form ref. 64. One AWATAR channel is defined, including all wells and all transition structures. The kinetics resulting from example12.rrk is shown as lines in Fig. 10(b), alongside the corresponding experimental kinetics data (circles) reported in ref. 64. Notably, the excellent agreement between the simulated and experimental results is achieved by fitting activation energy and scaling of IR intensities to the kinetics data and Arrhenius plots of multiple data sets at different temperatures, see ref. 64 for details.

For a properly thermalized population, (*i.e.*, when the initial population matches the steady-state population at the temperature of the environment), the decay of the reactant is expected to be exponential, which appears linear in the semi-logarithmic plot in Fig. 10(b). This behaviour is indeed observed in the experimental kinetics, and also reproduced by the MEM simulation as shown in Fig. 10(b).

However, if the initial cluster temperature is lower than the environmental temperature, the cluster will heat up during the experiment, leading to a phenomenon known as induction delay. Initially, dissociation is partially suppressed due to the lower energy available in the cluster. Over time, as the population approaches the steady state, the expected linear behavior is reached. Such induction delays can be simulated in AWATAR by setting the initial temperature of the population in the parameter window. Fig. 10(c) illustrates simulated induction delays for initial temperatures of 200 K and 100 K, shown in green and purple, respectively. The induction delay results in reduced dissociation in the beginning. However, after a few seconds, the gradient of the reactant decay becomes identical for all three cases shown, indicating that the dissociation rate constant is ultimately independent of the initial cluster temperature, if extracted from the linear part of the kinetics in the semi-logarithmic plot. MEM simulations with AWATAR can help to verify that these conditions have actually been met in the experiment, or can guide the experimentalist in planning the experiment accordingly.

### 4.4. Steady state MEM

Besides the simulation of real time MEM, AWATAR can also simulate the steady state of a population, which is reached after some time if the environmental conditions stay constant. Steady state means, that energy distribution of the population as well as the amount of product formed during the finite time step Δ*t* is converged.

The file *example13.rrk* provides a steady state MEM calculation for CO_3_(H_2_O)_2_^−^ performed at two environmental temperatures, 288 K and 250 K. All parameters, wells and TSs are the same as in *example12.rrk* discussed in the previous chapter. The steady-state MEM parameters are set such, that the calculation runs in a comparably short time (see user manual for details). In this mode, the finite time step and convergence criterion are initially rough, but are refined iteratively during the calculation. The population is written regularly in the file *example13_pop.txt*, and the current status of the calculation is stored in a checkpoint file on a regular basis. The simulation finishes, once the population is converged at the smallest requested time step. The results panel contains MEM rate coefficients for all temperatures and for all defined channels. These rate coefficients can be plotted in an Arrhenius plot, and comparison with experimental data allows the determination of the activation energy and the scaling of IR intensities as described in detail in ref. 46 and 48.

**Fig. 11:**
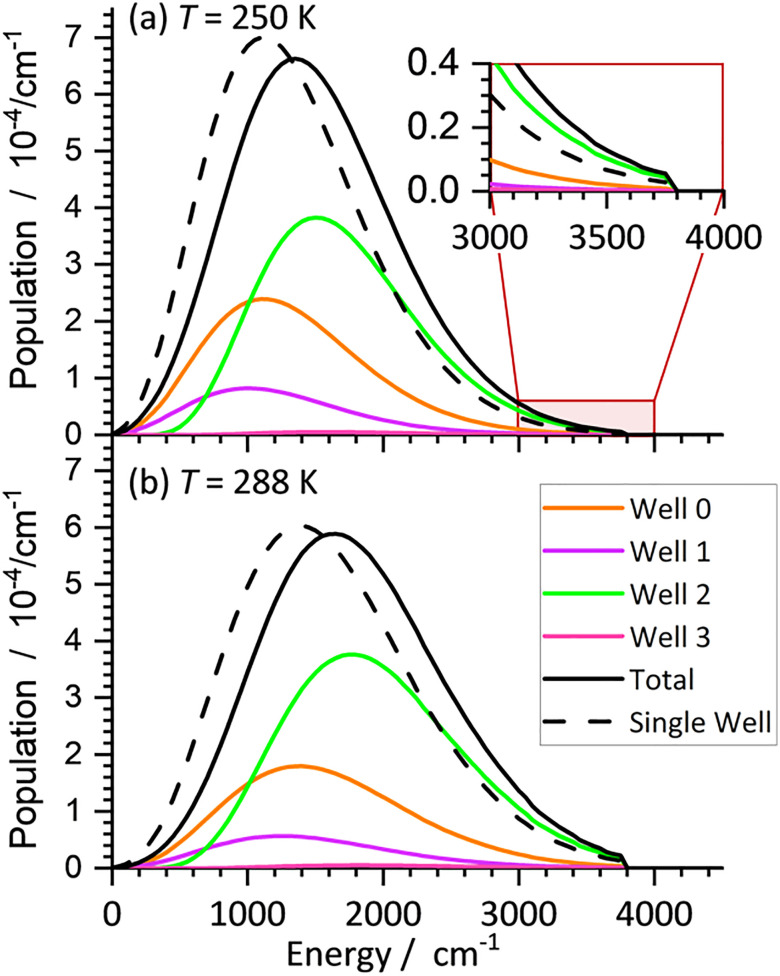
Converged populations of CO_3_(H_2_O)^−^, calculated with steady state MEM at environmental temperatures (a) *T* = 250 K and (b) *T* = 288 K using the AWATAR approach with all known wells. Note, that the most populated well (green) is not the global minimum (orange). The dashed line is the population of the single well approach.

Fig. 11 shows the resulting population for both calculated temperatures. Like the Ar_2_FeH^+^ case, the population sharply falls off above the energy of the lowest-lying transition structure. The total population is shown in black, while the coloured lines provide the contribution of the individual wells. Interestingly, the global minimum (orange) is not the most populated Well (green). This is because Well 2 has a higher density of states for *E* > 1000 cm^−1^. Therefore, the total population is shifted toward higher energies due to the inclusion of all wells in the simulation, and the average dissociation rate is higher compared to the single well model, shown with a dashed line for comparison. Inclusion of all wells in the MEM can thus lead to an improved determination of the activation energy,^48,64^ because it provides a more realistic representation of the experiment than single-well simulations.

## 5. Summary and outlook

We hope that the examples provided here illustrate the added value that unimolecular statistical rate theory can provide for scientists working on gas-phase reactions. The examples shown here are taken from typical mass spectrometry settings. Modern techniques like IRMPD of trapped ions may benefit from a quantitative simulation of the process in real time. It is obviously valuable to know the timescale on which dissociation of a specific molecular or cluster ion can be expected. AWATAR1.0 provides this information with relatively little extra effort, if a quantum chemical calculation of the system is available.

Numerous desirable extensions to the code can be envisioned, such as the inclusion of quantum state models for anharmonic oscillators, collisional energy exchange in addition to radiative processes, or proper thermalization of ions and neutrals in a reactive collision, to name just a few. Any update of the code will refer to this tutorial review and will be released on Github and ZENODO. We hope that AWATAR1.0 helps to improve our understanding of the interplay of radiative and reactive processes on a molecular scale.

## Author contributions

Magdalena Salzburger: conceptualization (supporting), data Curation (lead), formal analysis (lead), investigation (lead), methodology (supporting), software (equal), validation (equal), visualization (lead), writing – original draft (lead), writing – review and editing (equal). Marc Reimann: formal analysis (supporting), funding acquisition (lead), investigation (supporting), software (supporting), writing – review and editing (lead). Jessica C. Hartmann: formal analysis (supporting), investigation (supporting), visualization (supporting), writing – review and editing (supporting). Milan Ončák: conceptualization (supporting), funding acquisition (lead), investigation (supporting), methodology (equal), software (supporting), supervision (equal), validation (equal), writing – review and editing (supporting). Martin K. Beyer: conceptualization (lead), data Curation (equal), formal analysis (supporting), funding acquisition (lead), investigation (equal), methodology (lead), software (lead), supervision (lead), validation (equal), visualization (supporting), writing – original draft (lead), writing – review and editing (equal).

## Conflicts of interest

There are no conflicts of interest to declare.

## Supplementary Material

CP-028-D6CP00705H-s001

CP-028-D6CP00705H-s002

CP-028-D6CP00705H-s003

CP-028-D6CP00705H-s004

## Data Availability

Example files are available as SI. Further background and annotations are provided in the SI. Supplementary information: detailed description of the MEM calculation; sample input files; discussion of BS-Quantum parameter; comments on input files. See DOI: https://doi.org/10.1039/d6cp00705h. The AWATAR 1.0 source code and executable, together with quickstart guide, user manual and sample input files, is published on Github and ZENODO, https://doi.org/10.5281/zenodo.19915221.
