# Supplementary figures and images for: A practical guide to RRKM theory, its simplified multi-well version AWATAR and master equation modelling of radiative processes
Source: Phys Chem Chem Phys. 2026 Apr 30;28(20):12172–87. doi: 10.1039/d6cp00705h (PMC13158931; doi:10.1039/d6cp00705h)

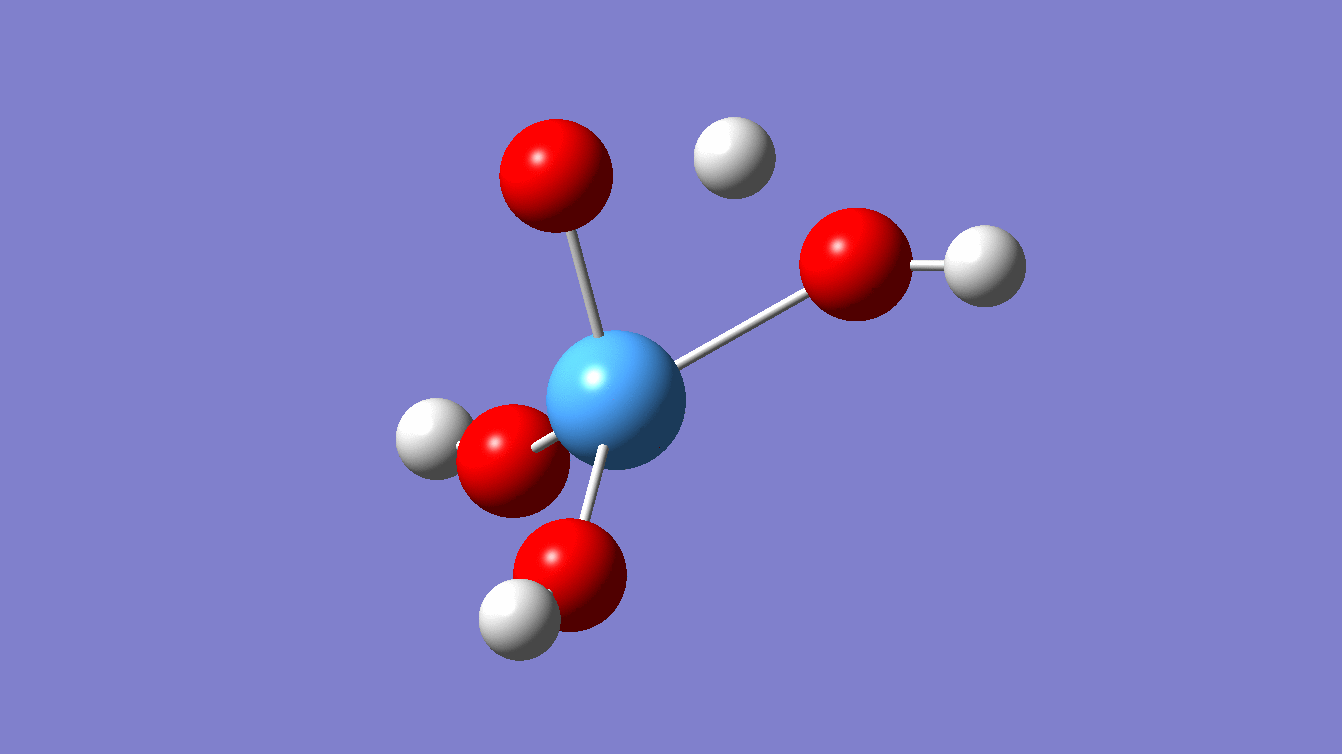

Supplement: CP-028-D6CP00705H-s001 [file CP-028-D6CP00705H-s001.gif]

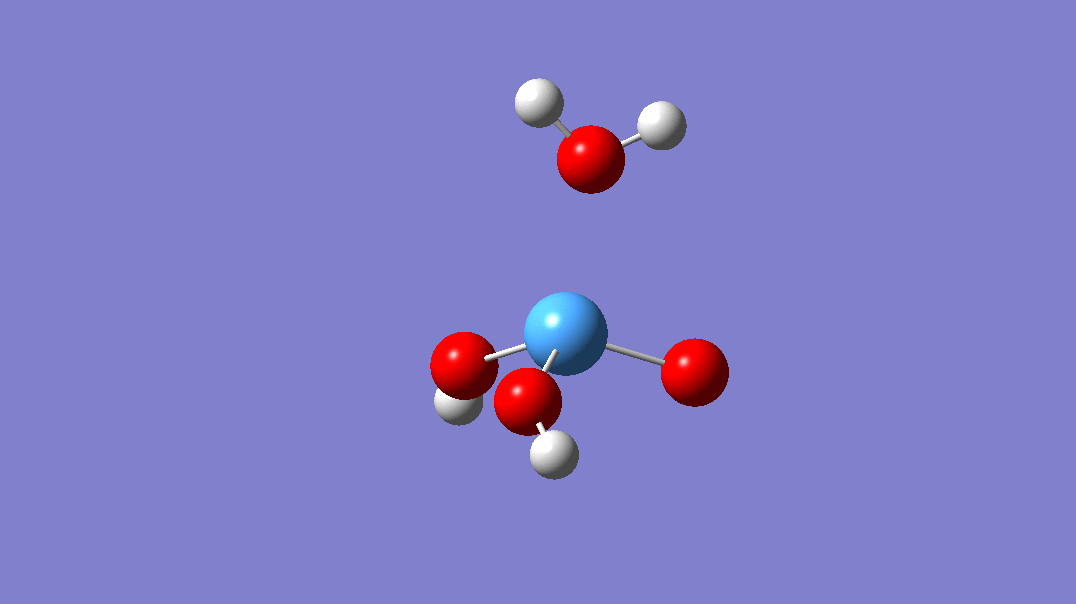

Supplement: CP-028-D6CP00705H-s002 [file CP-028-D6CP00705H-s002.gif]
