# Supplementary material for: A practical guide to RRKM theory, its simplified multi-well version AWATAR and master equation modelling of radiative processes
Source: Phys Chem Chem Phys. 2026 Apr 30;28(20):12172–87. doi: 10.1039/d6cp00705h (PMC13158931; doi:10.1039/d6cp00705h)
Supplement: CP-028-D6CP00705H-s003 [file CP-028-D6CP00705H-s003.pdf]

Supplementary Information (SI) for Physical Chemistry Chemical Physics.
This journal is © the Owner Societies 2026

Tutorial Review:

A Practical Guide to RRKM Theory, its Simplified Multi-Well Version AWATAR and Master Equation Modelling of Radiative Processes

*Magdalena Salzburger, Marc Reimann, Jessica C. Hartmann, Milan137Ončák, Martin K. Beyer**

Universität Innsbruck, Institut für Ionenphysik und Angewandte Physik, Technikerstrasse 25, 6020 Innsbruck, Austria

Email: martin.beyer@uibk.ac.at

Electronic Supplementary Information (ESI)

Comments on Example Files

example1.rrk

Two wells and one transition structure are present which represent $Ta(OH)_4^+$, $TaO(OH)_2(H_2O)^+$ and TS1 from Figure 1. Further comments are placed in the Well and TS panels.

In the **Parameters** panel, changes from the default values are typeset in **bold**:

```
;General Parameters:
     The filename is:                                     example1
     Frequency Scale Factor:                             0.96  ; used in reading Gaussian Data
     Energy range of population in cm-1:                  40000
     Bin size in cm-1:                                    20
     Energy quantum for Beyer/Swinehart in cm-1:          1
     Print lines where results are zero (YES/NO):        YES   ; option for RRKM Rate Constants
and SoS
```

The energy range was expanded to 40000 cm^{-1}, because the energy of TS1 is close to 20000 cm^{-1}. This way, the plots in Figures 2 and 3 cover a meaningful range. The bin size was reduced to 20 cm^{-1}, so that the initial non-monotonic rise of the DoS near the threshold becomes visible.

```
;Reaction Channels:
    Channel 0 Well 0 TS 0          ; Isomerization forward via proton transfer Ta(OH)4+ ->
 TaO(OH)2(H2O)+
    Channel 1 Well 1 TS 0          ; Isomerization backward TaO(OH)2(H2O)+ -> Ta(OH)4+
```

Two reaction channels are defined. To keep track of the calculations and results, it is helpful to describe the channel definition in a comment, starting with a semicolon ";".

The **Results** panel of the RRKM calculation starts with a summary of relevant energies for the reaction channels:

```
Channel 0:     Well Energy:   0.00 cm-1             TS Energy:     19453.15 cm-1
Activation Energy:        19453.15 cm-1
Channel 1:     Well Energy:   10597.18 cm-1         TS Energy:     19453.15 cm-1
Activation Energy:        8855.97 cm-1
```

To correlate the channels with the wells and transition structures, refer to the parameter window. The activation energy is the difference between TS Energy and Well Energy. It is not directly used in the calculation, but provided to guide intuition. In the present case, with the same TS for both channels, the channel with the lower barrier has significantly higher RRKM rate constants.

At the bottom of the panel, the relative energies of all Wells and TSs are summarized:

```
Further Information:

          Degeneracy:       Relative Energy:
Well 0:      1              0.0000 cm-1
Well 1:      1              10597.1816 cm-1
TS 0:        1              19453.1504 cm-1
```

This information is redundant, but it may be helpful for troubleshooting, especially in view of the various options to define energies in the Well*x*, TS*y* and Parameters panels.

The DoS shown in Figure 3a was also calculated with example1.rrk.

example2.rrk

This example illustrates the definition of tight and loose transition structures. Well 0 and Well 1 represent I1 and I2 from Figure 1, while TS 0 and TS 1 are the tight and loose TS, respectively. Results in Figure 4 are generated using this file. The RRKM rate constants were calculated and multiplied with the probability of I2, calculated via the DoS of Well 0 and Well 1.

The tight TS 0 is constructed from the same file as Well 1. By visual inspection of the vibrational modes, we identified the 431.1762 cm^{-1} vibration as the dissociative mode, see animation2.gif. This mode is commented out in panel **TS 0**:

```
  374.2972    vib  359.3253
  412.8913    vib  396.3756
;  431.1762    vib  413.9292  ; reaction coordinate / dissociative mode
  436.7108    vib  419.2424
  533.7026    vib  512.3545
```

The loose TS 1 is generated as described in detail in the manual, reading the two fragments with the buttons “Load loose TS Fragment 1” and “Add loose TS Fragment 2”.

In the panel **Parameters**, two channels are defined to compare the effect of tight vs. loose TS:

```
;Reaction Channels:
  Channel 0 Well 1 TS 0 ; Dissociation tight TS from Well 1, H2O loss from 1st solvation shell
  Channel 1 Well 1 TS 1 ; Dissociation loose TS from Well 1, H2O loss from 1st solvation shell
```

TS 0 still has the same energy as Well 1, which does not make sense. To make sure that both TS have identical energy, we define it in the panel **Parameters**, using the zero-point corrected dissociation energies:

```
;Fit parameters:
; 1) Adapt Energies
    Absolute energy of: TS 0, TS 1 is: 29842.5  ; define TS energy in cm-1 including ZPE relative
                         to global minimum - This overwrites the TS energy defined by TS-Panel.
;      Offset to Energy of all TS in cm-1:                 100.00          ; Adds Offset to
all transition states.
;      Offset to Energy of: TS 0, TS 1, TS 7 in cm-1:       100.00          ; Adds Offset to
the defined transition states
```

example3.rrk

The third example is from our publication where we introduced AWATAR to describe the unimolecular dissociation of sodium chloride clusters.[1] We describe the dissociation of $(NaCl)_4Na^+$ with loose transition states. Classic RRKM results with one Well dissociating via one loose TS are shown in Figure 5 (d), the corresponding AWATAR values are shown in Figure 5 (e). We identified 9 isomers of the cluster, Well 0 – Well 8 in **example3.rrk**. These can dissociate via 16 loose TS into fragments of different size.

Once all these isomers of reactants and products are calculated, it is straightforward to setup the AWATAR job to calculate the rate constants. After reading the Wells and TSs, all that remains to be done is the definition of the reaction channels. For the four different fragment sizes, we defined one AWATAR channel each. For comparison, we defined classic RRKM channels.

```
;Reaction Channels:

; AWATAR Channels
     Channel 0 Well all TS 0 1 2 3 4 5 6 7       ; n=4
     Channel 1 Well all TS 8 9                   ; n=3
     Channel 2 Well all TS 10 11 12              ; n=2
     Channel 3 Well all TS 13 14 15 16           ; n=1

; Classic RRKM channels with one well and one TS:

     Channel 4 Well 0 TS 0                       ; n=4
     Channel 5 Well 0 TS 8                       ; n=3
     Channel 6 Well 0 TS 10                      ; n=2
     Channel 7 Well 0 TS 13                      ; n=1
```

example4.rrk

Here back dissociation and product formation are described as loose TS 0 and TS 1, respectively, while Well 0 represents the collision complex, reflected in the **Parameters** panel.

```
;Reaction Channels:
    Channel 0 Well 0 TS 0    ; k_b
    Channel 1 Well 0 TS 1    ; k_p
```

example5.rrk

The only difference to example4.rrk is the adjustment of the TS 1 energy to match the experimental proton affinities.

```
;Fit parameters:
; 1) Adapt Energies

        Absolute energy of: TS 1,          is:      7555.4685         ; Energy Calculated from
experimental proton affinities such, that the enthalpy difference to TS0 matches the difference
in proton affinities.
```

example6.rrk

Here we added higher lying isomers of the products and collision complex, TS 2–3 and Well 1–4, respectively. For the analysis of back and forward reaction, we defined AWATAR channels.

```
;Reaction Channels:
    Channel 0 Well all TS 0 2   ; k_b
    Channel 1 Well all TS 1 3   ; k_p
```

The energies of all TSs is adjusted to reflect experimental thermochemistry.[2]

```
;Fit parameters:

      Absolute energy of: TS 1          is:     7555.4685         ; Energy Calculated …
      Absolute energy of: TS 2          is:     19885.436         ; Energy Calculated …
      Absolute energy of: TS 3          is:     9695.4493         ; Energy Calculated …
```

example7.rrk

A simple example of real-time MEM. We have only one well, and no TS. We can still define an AWATAR channel in the **Parameters** panel. Since the sum of states in this case is 0 at all energies, RRKM rate constants are also 0 for all energies.

```
;Reaction Channels:
     Channel 0 Well all TS all
```

This may look strange, but it is useful to model the internal energy of Well 0 in real time. A warning is displayed when you press “Start MEM”, but the program runs nevertheless.

To save computing time, we limit the energy range to 15000 cm^{-1}. For smooth populations, we set “Bin size” to 20 cm^{-1}. During the simulation run, population is written to the file example7_pop.txt.

```
;General Parameters:
     The filename is:                             example7 ;C6H2I3COO- cooling down from 500 K
     Frequency Scale Factor:                       0.96  ; used in reading Gaussian Data
     Energy range of population in cm-1:           15000
     Bin size in cm-1:                             20
     Energy quantum for Beyer/Swinehart in cm-1:   1
     Print lines where results are zero (YES/NO):  YES ; option for RRKM Rate Constants and SoS
```

For real time MEM, we have to set a couple of parameters in the General MEM and Real time MEM sections.

```
;Definitions for master equation modeling (MEM):
;General MEM parameters:
;   BIRD parameters:
       Number of temperatures in loop:                    1
       Manual list for temperatures in loop:              300  ;Define Temperatures …
       Loop starts with temperature in K:                 200
       Loop ends with temperature in K:                   300
       Proportion on solid angle of ICR cell window:      0.0
       Temperature of ICR cell window in K:               288
;  IRMPD parameters:
       Rate of Photon absorption for Well all in s-1:     0
;      Rate of Photon absorption for Well 0 3 7 in s-1:   0
```

```
      IRMPD photon energy in cm-1:                        0
   Finite time step parameter:                            0.000001 ; Used for real time MEM
;Real time MEM parameters:
      Run MEM in real time (YES/NO):                      YES
      Total simulation time in seconds:                   10
      Interval for data point export in seconds:       0.01  ; reactant and product intensity
      Export population after n data points: 10  ; population written after 10 data points each
      Initial Cluster Temperature in K:                   500    ; active if non-zero
      Photon energy at t = 0 in cm-1:                     0
```

The first temperature defines the radiation field during MEM, a black-body radiation field at the given temperature, here 300 K. The finite time step is relatively small. When you run the simulation for the first time, make the step a factor of 100 bigger to reduce computing time, otherwise the wait for the next data point becomes very long. For data production, it is advisable to choose small enough time steps so that the finite elements simulation provides smooth results.

The “YES” tells AWATAR to execute real-time MEM upon pressing the “Start MEM” button.

Total simulation time is 10 s in real time. The export interval determines how many data points are printed on the Console window and in the results panel, in this case 10 s / 0.01 s = 1000. If you want fewer data points, increase the interval.

The cluster temperature is set to 500 K at *t* = 0 s. AWATAR calculates a Boltzmann distribution and writes it to the population file defined above, before the first time step is executed. Therefore, the first population you find in a population file is the Boltzmann distribution without any reactions/dissociations. This should be kept in mind when interpreting the *t* = 0 s population. If you need an early population that includes dissociation, run a very short real-time MEM and take the second population from the file.

The results window contains information on reactant and product formation, as well as the mean energy of the population, averaged over all wells. Here we have no reaction, so the relevant result is mean energy vs. time. You can now copy the content of the window via the mouse buffer to a spreadsheet program and display the results graphically. Note that the block with the real-time MEM is followed by RRKM rate constants, density of states and sum of states for full documentation of the simulation and quality control.

```
;Master Equation Modeling in Real Time

Temperature 300.00 K
          Time          Reactant         Channel__0       Mean_Energy
           (s)                                                (cm-1)
  0.000000e+000      1.000000e+000      0.000000e+000      5.149985e+003
  1.000000e-002      1.000000e+000      0.000000e+000      5.079322e+003
  0.200000e-001      1.000000e+000      0.000000e+000      5.011254e+003
  0.300000e-001      1.000000e+000      0.000000e+000      4.945651e+003
  0.400000e-001      1.000000e+000      0.000000e+000      4.882390e+003
  0.500000e-001      1.000000e+000      0.000000e+000      4.821355e+003
  0.600000e-001      1.000000e+000      0.000000e+000      4.762439e+003
```

```
  0.700000e-001       1.000000e+000       0.000000e+000       4.705541e+003
       …
```

example8.rrk

The only difference to example7.rrk is the initial cluster temperature, which is set to 100 K to simulate radiative heating.

example9.rrk

This is again based on example7.rkk, but now the initial temperature parameter is set to 0, which means the temperature of the environment is used for the initial Boltzmann distribution. We simulate that a photon was absorbed at t = 0 s. The photon energy of 3091.4259 cm^{-1} corresponds to absorption into the symmetric C-H stretching mode of the molecular ion. This shifts the initial 300 K population to higher energies. The simulation illustrates the thermalization of the system after absorption of such an IR photon in a 300 K black-body radiation environment without collisions.

```
;Real time MEM parameters:
       Run MEM in real time (YES/NO):                      YES
       Total simulation time in seconds:                   10
       Interval for data point export in seconds:          0.01
       Export population after n data points:              10              ; population …
       Initial Cluster Temperature in K:                    0   ; active if non-zero …
       Photon energy at t = 0 in cm-1:  3091.4259      ; Photon energy corresponds to symmetric
                                                          C-H stretching mode
```

Of course, higher values can be chosen to simulate colling after absorption of a UV/VIS photon. One can in this case include higher lying electronic states as additional wells, if all wells are connected via conical intersections, and dissociation or isomerization channels. Note that the relevant processes will be much faster at higher energies, and the total simulation time and time step must be chosen accordingly.

example10.rrk

Again based on example7.rrk, heating by a constant flux of IR photons is simulated. To this end, we use the section “IRMPD parameters:” in the **Parameters** panel. We have the option to set a rate of photon absorption for all wells or for groups of wells. The line with the group of wells may be included multiple times to reflect different IR absorption cross sections for different isomers. Here, we have only one well, and assume a rate of photon absorption of

1 s^{-1}. The IR laser source provides 1694.4417 cm^{-1} photons. Comments have been removed to improve readability.

```
;  IRMPD parameters:
       Rate of Photon absorption for Well all in s-1:       1
;      Rate of Photon absorption for Well 0 3 7 in s-1:     0
       IRMPD photon energy in cm-1:                         1694.4417
   Finite time step parameter:                              0.00001
;Real time MEM parameters:
       Run MEM in real time (YES/NO):                       YES
       Total simulation time in seconds:                    10
       Interval for data point export in seconds:           0.01
       Export population after n data points:               10
       Initial Cluster Temperature in K:                    0
       Photon energy at t = 0 in cm-1:                      0
```

example11.rrk

Here we apply real-time MEM to simulate experimental IRMPD data of Ar_2FeH^+. Due to the small binding energy of Ar in Ar_2FeH^+, an energy range of 4000 cm^{-1} is sufficient:

```
     Energy range of population in cm-1:                   4000
     Bin size in cm-1:                                     20
```

In the MEM parameter section, we invoke several parameters to simulate IRMPD at two different environmental temperatures, 80 K and 300 K. The proportion of the ICR cell window that is exposed to the water-cooled UVH tube is included. A rate of photon absorption of 0.34 s^{-1} at 1855 cm^{-1} was found to provide the best match of the simulation with experimental IRMPD kinetics. We assume that Ar_2FeH^+ ions leave the laser vaporization source with vibrational excitation matching the source temperature.

```
;Definitions for master equation modeling (MEM):
;General MEM parameters:
;   BIRD parameters:
       Number of temperatures in loop:                      2
      Manual list for temperatures in loop:                 80 300
       Loop starts with temperature in K:                   200
       Loop ends with temperature in K:                     300
       Proportion on solid angle of ICR cell window:        0.002
       Temperature of ICR cell window in K:                 288
;  IRMPD parameters:
       Rate of Photon absorption for Well all in s-1:         0.34
;      Rate of Photon absorption for Well 0 3 7 in s-1:       0
       IRMPD photon energy in cm-1:                         1855 ; photon energy in experiment
   Finite time step parameter:                              0.00001
;Real time MEM parameters:
       Run MEM in real time (YES/NO):                       YES
       Total simulation time in seconds:                    10
       Interval for data point export in seconds:           0.1
       Export population after n data points:               10
       Initial Cluster Temperature in K:            300   ; temperature of ion source
       Photon energy at t = 0 in cm-1:                      0
```

To reach agreement with experiment, additional parameters must be adjusted. Note that AWATAR does not provide a fit function to adapt the parameters of real-time MEM to experimental data. The fitting has been done by trial-and-error.

```
;Fit parameters:
; 1) Adapt Energies
      Absolute energy of: TS 0     is:     2423    ; from high-level quantum chemistry
; 2) Adapt infrared intensities
```

```
      Scaling of IR intensities:          4.797   ; Fitted to experimental BIRD kinetics
```

The **Results** panel contains data on product formation in column **Channel__0**.

```
Temperature 80.00 K
           Time           Reactant         Channel__0        Mean_Energy
            (s)                                                  (cm-1)
  0.000000e+000     1.000000e+000     0.000000e+000     8.368272e+002
  1.000000e-001     0.972714e-000     0.272858e-001     7.890113e+002
  0.200000e-000     0.952553e-000     0.474474e-001     7.565158e+002
  0.300000e-000     0.933892e-000     0.661082e-001     7.284786e+002
  0.400000e-000     0.916621e-000     0.833785e-001     7.044786e+002
       …
```

example12.rrk

Another example based on previously published data.[3] We simulate BIRD of $CO_3^-(H_2O)_2$, as illustrated in Figure 10. Real-time MEM yields first-order kinetics as observed experimentally. Again, suitable energy range and Bin size are chosen.

```
    Energy range of population in cm-1:                 10000
     Bin size in cm-1:                                   50
```

Real-time MEM parameters are defined as explained above, e.g. example7.rrk.

```
;Definitions for master equation modeling (MEM):
;General MEM parameters:
;   BIRD parameters:
       Number of temperatures in loop:                    1
      Manual list for temperatures in loop:              288
       Loop starts with temperature in K:                 200
       Loop ends with temperature in K:                   300
       Proportion on solid angle of ICR cell window:      0.002
       Temperature of ICR cell window in K:               288
;  IRMPD parameters:
       Rate of Photon absorption for Well all in s-1:     0
;      Rate of Photon absorption for Well 0 3 7 in s-1:   0
       IRMPD photon energy in cm-1:                       0
   Finite time step parameter:                            0.00001
;Real time MEM parameters:
       Run MEM in real time (YES/NO):                     YES
       Total simulation time in seconds:                  10
       Interval for data point export in seconds:         0.01
       Export population after n data points:             100
       Initial Cluster Temperature in K:                  0
       Photon energy at t = 0 in cm-1:                    0
```

Energies are adapted to best available values. IR intensities are scaled with factor obtained previously.[3] Note that binding energies and IR intensity scaling factor cannot be obtained from fitting a single BIRD kinetics. Temperature dependent experiments are necessary, as detailed before.[3,4]

```
;Fit parameters:
; 1) Adapt Energies
       Absolute energy of: TS 0      is:    3762.00    ; 45 kJ/mol³
       Absolute energy of: TS 1      is:    3845.00    ; calculated with CCSD(T)/aug-cc-pVDZ
; 2) Adapt infrared intensities
       Scaling of IR intensities:          1.3           ; previous fit to Arrhenius plot³
```

For the results shown in Figure 10c, the initial cluster temperature was set to 200 K and 100 K, respectively.

Example13.rrk

Based on example12.rrk, this example illustrates the use of iterative MEM. The changes to switch to iterative MEM are straightforward. The simulation is for two temperatures, which are defined in the manual list. For use of the loop parameters, see manual. “NO” directs the program to steady state MEM, which means, that the goal of the simulation is to calculate the final converged population at a steady state. AWATAR narrows down iteratively the used time steps and convergence criterion, if the button “Iterative MEM” is pressed. (For full control of this procedure, the narrowing down could alternatively be performed manually, using the buttons “Start MEM and “Continue MEM” instead, see manual for details.) The relevant parameters for steady state MEM, using the button “Iterative MEM”, are the “Minimum time step at 300 K” and the “Minimum number of iterations”. See manual for more details on the procedure of iterative MEM and influence of the parameters on the simulation.

```
;Definitions for master equation modeling (MEM):
;General MEM parameters:
;   BIRD parameters:
       Number of temperatures in loop:                    2
      Manual list for temperatures in loop:              288 250  ;Define Temperatures in K
       Loop starts with temperature in K:                 200
       Loop ends with temperature in K:                   300
       Proportion on solid angle of ICR cell window:      0.002
       Temperature of ICR cell window in K:               288
;  IRMPD parameters:
       Rate of Photon absorption for Well all in s-1:     0
;      Rate of Photon absorption for Well 0 3 7 in s-1:   0
       IRMPD photon energy in cm-1:                       0
   Finite time step parameter:                            0.00001
;Real time MEM parameters:
       Run MEM in real time (YES/NO):                     NO
       Total simulation time in seconds:                  10
       Interval for data point export in seconds:         0.01
       Export population after n data points:             100
       Initial Cluster Temperature in K:                  0
       Photon energy at t = 0 in cm-1:                    0
;Steady state MEM parameters:
       Minimum time step at 300 K:   8     ; translates to 10E-8 s, replaces finite time step
       Minimum number of iterations:     400            ; Used for Iterative MEM
       Convergence parameter:            0.0001        ; for Start MEM and Continue MEM
```

The **Results** panel of iterative MEM provides temperature-dependent BIRD rate coefficients and their branching over the different reaction channels, followed by diagnostics output. At the very end, the last timestep and the final convergence parameter are given.

```
Master Equation Modeling for the calculation of a steady state

 Temperature   1/Temperature  rate_coefficient  ln(rate_coefficient/(s-1))  Channel__0
         (K)           (K-1)             (s-1)                                   (s-1)
     288.000      0.00347222        0.07800657                 -2.55096221  0.07800657
     250.000      0.00400000        0.01406208                 -4.26427364  0.01406208

       …

Calculated with Iterative MEM.    Last timestep(at 300K): 0.000000010000000 s
Reached convergence: 0.000009999999747   Last Number of Iterations: 2000

Populations in file example13_pop.txt
```

Details and Examples on the Choice of BS-Quantum

The density of states should not change significantly when BS-Quantum is modified. A value of BS-Quantum = 1.0 cm^{-1} usually works well, but small systems may require significantly larger values to avoid unphysical behaviour. Conversely, smaller values may be chosen for large systems with small rotational constants and low-lying vibrational modes. It is advised to modify this parameter and verify that the results are robust, *i.e.* they do not change significantly when BS-Quantum is modified.

The underlying reason is that the Beyer-Swinehart direct count algorithm needs to find a quantum state that precisely matches the total energy E. If the energy eigenvalues of the individual degrees of freedom are given with a high precision, it is highly unlikely that the sum of a few such numbers precisely equals E. In general, a coarser graining of the energies gives a higher number of combinatorial options to match E. The density of states can still remain constant, because we divide the number of combinations by BS-quantum to obtain this value. For the sum of states of transition structures, we also get more options for a specific energy in the reaction coordinate with coarser graining / higher value of BS-quantum, but at the same time, we sum over fewer such values to obtain the sum of states. In both cases, there is a range of values for BS-quantum for which density and sum of states are more or less constant.

These arguments also illustrate that the density and sum of states obtained in this way are not highly precise values, but rather the best possible compromise we can get.

Additional Figures

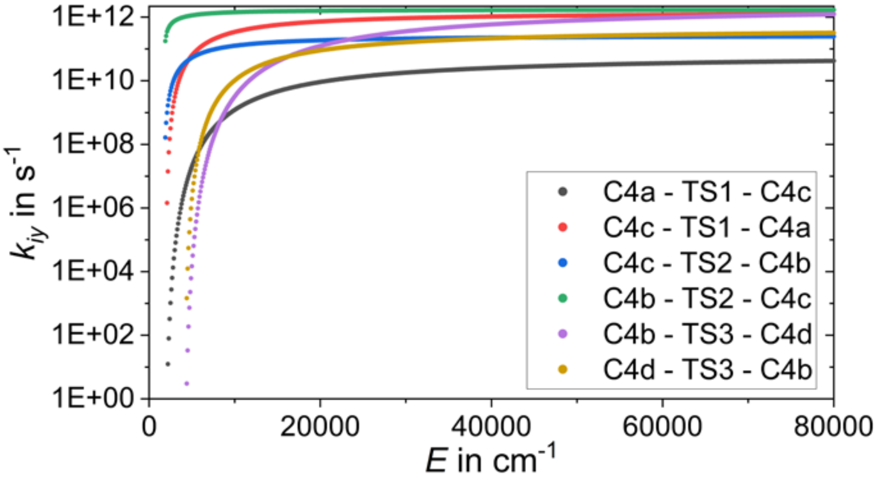

Figure S1: RRKM rate constants k_{iy} as a function of energy E for the isomerization between the isomers shown in Figure 1.

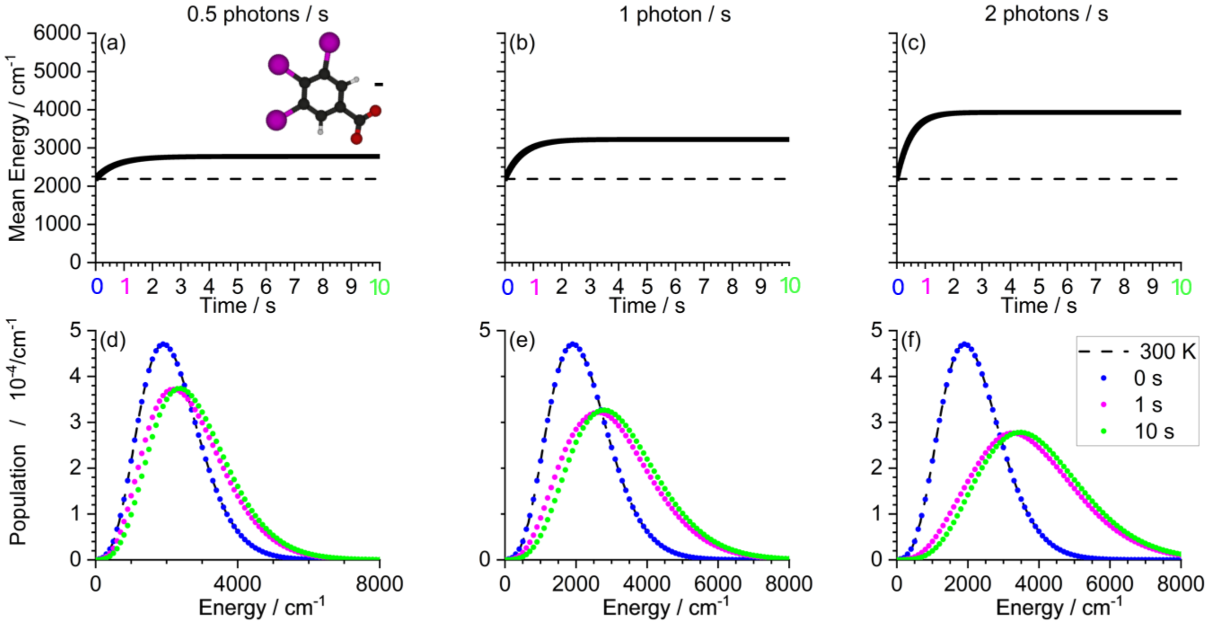

Figure S2: MEM of $C_6H_2I_3COO^-$ simulating changes in internal energy under the influence of an IRMPD laser with different rates of photon absorption. Note, that the mean energy rises more, if the rate of photon absorption is higher. In addition, the population gets broader with increasing rate of photon absorption.

Detailed description of the MEM calculation

The Master Equation Modeling (MEM) simulates the time evolution of a population by accounting for photon absorption, photon emission, and dissociation. In AWATAR, the MEM calculation proceeds as follows:[6]

The process begins by assuming that the internal energy of the population follows a Boltzmann distribution at an initial temperature T_i which is defined in the parameters panel under the section "BIRD parameters" or by the parameter "Initial Cluster Temperature in K". This energy distribution is divided into small energy bins b with a width ΔE as specified by the parameter "Bin size in cm-1". The initial population P_{ib} for isomer i and bin b is given by

$$P_{ib} = \frac{\rho_{ib}\, \mathrm{e}^{-E_b/(k_\mathrm{B}\, T_i)}}{\sum_j \sum_n \rho_{jn}\, \mathrm{e}^{-E_n/(k_\mathrm{B}\, T_i)}} \tag{1}$$

Here, k_B is Boltzmann's constant, E_b is the energy of bin b relative to the ground state of the global minimum, given by $E_b = b\Delta E$ and ρ_{ib} is the density of states of isomer i and bin b. The denominator in eq. (1) normalizes the population, with the sums over j and n representing the sums over all isomers and bins, respectively.

Next, the time evolution of this initial population is simulated. Photon absorption and emission shift the population toward higher or lower energies, respectively. For each transition from bin b to bin c, the rates for absorption, $k_{\mathrm{abs},ibc}$ (if $c > b$) or emission $k_{\mathrm{em},ibc}$ (if $c < b$), are calculated as follows:

$$k_{\mathrm{abs},ibc} = \sum_q \sum_m \frac{\rho_{mib}}{\rho_{ib}} (m+1) \left(r\, B_{12,iq}\, d_{q,T_\mathrm{E}} + (1-r)\, B_{12,iq}\, d_{q,T_\mathrm{e}}\right) \tag{2}$$

$$k_{\mathrm{em},ibc} = \sum_q \sum_m \frac{\rho_{mib}}{\rho_{ib}}\, m \left[\left(r\, B_{21,iq}\, d_{q,T_\mathrm{E}} + (1-r)\, B_{21,iq}\, d_{q,T_\mathrm{e}}\right) + A_{21,iq}\right] \tag{3}$$

In these equations, the sum over q includes all vibrational modes q whose energy $h\nu_q$ matches the energy gap between bin b and bin c, satisfying the condition

$$-\frac{1}{2}\Delta E \leq h\nu_q - |E_c - E_b| < \frac{1}{2}\Delta E \qquad (4)$$

In eq. (2) and eq. (3), the sum over m accounts for all energetically accessible vibrational quantum numbers m of mode q. Each possibility is weighted by its probability, given by ρ_{mib}/ρ_{ib} , the proportion of ρ_{mib}, the density of states with m times populated vibrational mode q, to the total density of states ρ_{ib} of the given bin and isomer. The factors m and (m+1) reflect the increased transition dipole moment at higher vibrational excitations in the harmonic approximation, which enhances the rates. Furthermore, r is the solid angle covered by the temperature of the environment T_E, while the remaining solid angle (defined by the parameter "Proportion on solid angle of ICR cell window") is at a temperature T_e, (defined by the parameter "Temperature of ICR cell window in K"). The terms d_{q,T_E} and d_{q,T_e} are the blackbody radiation densities at frequency ν_q for the respective temperatures.

The Einstein coefficients $A_{21,iq}$, as well as $B_{12,iq}$, and $B_{21,iq}$ multiplied by the respective radiation density are calculated as:

$$A_{21,iq} = \frac{8\,\pi\,c\,\tilde{\nu}_{iq}^2 I_{iq}\,s}{N_A} \qquad (5)$$

$$B_{12,iq}\,d_{q,T} = B_{21,iq}\,d_{q,T} = \frac{8\,\pi\,c\,\tilde{\nu}_{iq}^2\,I_{iq}\,s}{N_A\left(e^{\tilde{\nu}_{iq}\,h/(k_B\,T)} - 1\right)} \qquad (6)$$

Here, c is the speed of light, $\tilde{\nu}_{iq}$ is the wavenumber of the vibrational mode q (in m^{-1}) for isomer i, I_{iq} is the corresponding IR intensity (in m/mol), s is the scaling factor for infrared intensities defined by the parameter "Scaling of IR intensities", accounting for example for anharmonicities, and N_A is Avogadro's constant. In eq. (6), T refers to either T_E or T_e, and h is Planck's constant. In addition to photon absorption and emission, dissociation is possible for all bins with internal energy exceeding the activation energy. The dissociation rate constant for bin b is denoted as $k_{diss,b}$. In the AWATAR approach, $k_{diss,b}$ is the same for all isomers, and calculated as follows:

$$k_{\text{diss},b} = \frac{\sum_y \sigma_{\text{trans},y} N_{yb}}{h \sum_j \sigma_{\text{well},j}\, \rho_{jb}} \tag{7}$$

Here, the numerator is the total sum of states at bin *b* of the dissociated cluster, including all transition states, while the denominator is Planck's constant *h* multiplied with the total density of states at bin *b* of the parent ion, including all isomers. N_{yb} is the sum of states of transition state *y*, ρ_{jb} is the density of states of isomer *j* and $\sigma_{\text{trans},y}$ and $\sigma_{\text{well},j}$ are degeneracies of transition state *y* and well *j*, respectively.

Once all rate constants are determined, the population change within a time step Δt is calculated. First, the population loss ΔP_{ib}^- is calculated for each isomer and bin. Therefore, an exponential decay function is used as it is better suited to cover many orders of magnitude:

$$\Delta P_{ib}^- = P_{ib}\,[1 - \mathrm{e}^{k_{\text{loss},ib}\,\Delta t}] \tag{8}$$

where $k_{\text{loss},ib}$ is the total loss rate constant from bin b:

$$k_{\text{loss},ib} = k_{\text{diss},b} + \sum_{c>b} k_{\text{abs},ibc} + \sum_{c<b} k_{\text{em},ibc} \tag{9}$$

Similarly also the population gain ΔP_{ib}^+ is calculated as:

$$\Delta P_{ib}^+ = \sum_{c<b} \frac{k_{\text{abs},icb}}{k_{\text{loss},ic}}\, \Delta P_{ic}^- + \sum_{c>b} \frac{k_{\text{em},icb}}{k_{\text{loss},ic}}\, \Delta P_{ic}^- \tag{10}$$

The total change in the population ΔP_{ib} for isomer i and bin b is then:

$$\Delta P_{ib} = -\Delta P_{ib}^- + \Delta P_{ib}^+ \tag{11}$$

Since dissociated clusters are no longer part of the population, the total population decreases over Δt. This decrease, ΔP is calculated as

$$\Delta P = \left| \sum_j \sum_n \Delta P_{jn} \right| .$$

The rate coefficient k_{MEM} is then determined as:

$$k_{\text{MEM}} = -\frac{\ln(1-\Delta P)}{\Delta t} \tag{12}$$

The population $P_{ib}(t + \Delta t)$ of time t + Δt is given by

$$P_{ib}(t + \Delta t) = \frac{\sum_j (P_{jb}(t) + \Delta P_{jb})}{1 - \Delta P} \frac{\rho_{ib}}{\sum_j \rho_{jb}} \tag{13}$$

The first fraction in eq. (13) corresponds to the total population of bin b after timestep Δt, summed over all isomers j and normalized. The second fraction, $\rho_{ib}/\sum_j \rho_{jb}$ redistributes this total population among all isomers according to their density of states. The population after the next time steps is then calculated iteratively.

For steady state MEM, this cycle is repeated, until a steady state is reached, where the change of the normalized population becomes negligible and k_{MEM} converges. Initially, time step Δt and convergence criteria are rough, but they are refined iteratively until the minimum time step defined by the parameter “Minimum time step at 300 K” is reached.

References

(1) Hartmann, J. C.; Madlener, S. J.; van der Linde, C.; Ončák, M.; Beyer, M. K. Magic Cluster Sizes of Cationic and Anionic Sodium Chloride Clusters Explained by Statistical Modelling of the Complete Phase Space. *Phys. Chem. Chem. Phys.* **2024**, *26*, 10904-10918. DOI: 10.1039/D4CP00357H.

(2) Linstrom, P. J.; Mallard, W. G. *NIST Chemistry WebBook, NIST Standard Reference Database Number 69*. http://webbook.nist.gov/.

(3) Salzburger, M.; Ončák, M.; van der Linde, C.; Beyer, M. K. Simplified Multiple-Well Approach for the Master Equation Modeling of Blackbody Infrared Radiative Dissociation of Hydrated Carbonate Radical Anions. *J. Am. Chem. Soc.* **2022**, *144* (47), 21485–21493. DOI: 10.1021/jacs.2c07060.

(4) Dunbar, R. C. BIRD (Blackbody Infrared Radiative Dissociation): Evolution, Principles, and Applications. *Mass Spectrom. Rev.* **2004**, *23* (2), 127–158. DOI: 10.1002/mas.10074.

(5) Gilbert, R. G.; Smith, S. C. *Theory of Unimolecular and Recombination Reactions*; Physical Chemistry Texts; Blackwell Scientific Publications, 1990.

(6) Salzburger, M. Dissociation of hydrated carbonate radical anions and related clusters. Dissertation, Universität Innsbruck, Innsbruck, 2024. urn://nbn:at:at-ubi:1-154766 (accessed 2025-06-25).
